# Elevated 5-oxoproline levels and adverse outcomes in heart failure: Association with renal cortical OPLAH loss in a multi-comorbidity model

**DOI:** 10.1016/j.redox.2026.104354

**Published:** 2026-08-17

**Authors:** Antonio Esquivel-Gaytan, Ali A. Al-Mubarak, Oana Sorop, Daphne Merkus, Adriaan A. Voors, Herman H.W. Silljé, Dirk J. Duncker, Peter van der Meer, Nils Bomer

**Affiliations:** a Department of Experimental Cardiology, University Medical Center Groningen, University of Groningen, Groningen, the Netherlands; b Division of Experimental Cardiology, Department of Cardiology, Erasmus University Medical Center, Rotterdam, the Netherlands; c Institute for Surgical Research, Walter Brendel Center of Experimental Medicine, University Clinic Munich, LMU Munich, Munich, Germany; d German Center for Cardiovascular Research (DZHK), Munich Heart Alliance (MHA), Partner Site, Munich, Germany

**Keywords:** 5-oxoproline, Heart failure, Oxidative stress, Chronic kidney disease, Cardiorenal axis

## Abstract

**Background:**

Heart failure (HF) progression is closely linked to oxidative stress. 5-Oxoproline (5-OP), a product of glutathione degradation, is normally metabolized by 5-oxoprolinase (OPLAH) but accumulates when the gamma-glutamyl cycle is disrupted. Here, we investigated the clinical characteristics of circulating 5-OP, its proteomic correlates, and the associations to outcome in HF.

**Methods:**

In serum of 823 BIOSTAT-CHF patients, 5-OP was quantified by validated liquid chromatography–mass spectrometry and analyzed for associations with clinical outcomes. Proteomic correlates were identified across 355 OLINK proteins using stability selection with Minimax Concave Penalty regression. Mechanistic context was evaluated in a multi-comorbidity, large-animal cardio-kidney-metabolic (CKM) model with regional OPLAH assessment.

**Results:**

Higher 5-OP was associated with worse renal function (eGFR declining across 5-OP tertiles, 67.9 to 60.2 mL/min/1.73 m^2^; p = 0.0012) and higher all-cause mortality (HR 1.55, 95% CI 1.11–2.17, p = 0.010). Per SD increase in log-5-OP, risk for the 2-year composite endpoint increased (HR 1.27, 95% CI 1.05–1.53), with broadly similar associations across CKD strata (interaction p = 0.63). TGF-α was the most robust proteomic correlate (π = 0.70; empirical permutation p = 0.001). In CKM swine, circulating 5-OP was elevated and renal cortical OPLAH protein, but not cardiac OPLAH, was selectively reduced, consistent with a renal contribution to systemic 5-OP elevation.

**Conclusion:**

Circulating 5-OP identifies HF patients at higher risk and is robustly associated with TGF-α. In a translational swine model, selective loss of renal cortical OPLAH provides tissue context supporting a renal contribution to systemic 5-OP elevation in cardiorenal syndrome.

## Graphical abstract

**Figure:**
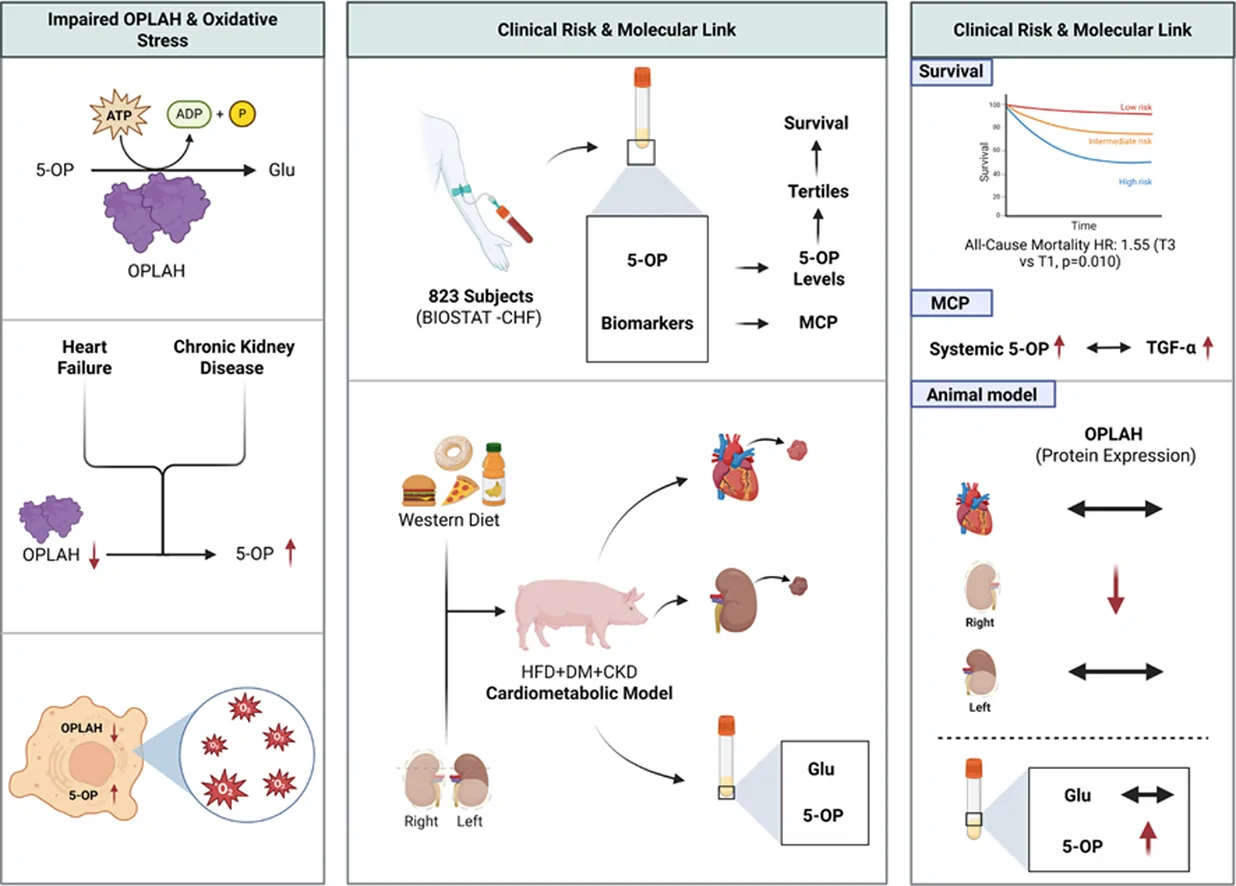

## 1 Introduction

Heart failure (HF) is a prevalent and complex clinical syndrome resulting from various structural or functional cardiac abnormalities that impair the heart's ability to circulate blood effectively [1,2]. One particularly challenging aspect of HF is its frequent association with kidney dysfunction, a condition known as cardiorenal syndrome (CRS). CRS arises from complex bidirectional interactions between the heart and kidneys, wherein dysfunction in one organ exacerbates dysfunction in the other [3,4]. In heart failure with preserved ejection fraction (HFpEF), renal impairment has been proposed not only as a comorbidity but as an upstream driver of systemic metabolic disturbances that contribute to inflammation and endothelial dysfunction, thereby contributing to myocardial remodeling [5,6]. However, the relationship between oxidative stress and CRS represents a critical yet poorly understood aspect of disease progression [7,8].

Oxidative stress plays a central role in the progression of both cardiac [9] and renal [10,11] failure pathophysiology. Maintaining the balance between reactive oxygen species (ROS) production and antioxidant defenses is critical for cellular homeostasis in both organs. When this balance is disrupted, excessive oxidative stress can trigger a cascade of events leading to either accelerated cardiac or renal dysfunction, or both [[7], [8], [9], [10]]. A crucial mechanism for maintaining cellular redox balance is the gamma-glutamyl cycle, which regulates the synthesis of glutathione (GSH), a powerful antioxidant [12] and has been shown to be reduced in HF [13]. One of the key enzymes in this cycle, 5-oxoprolinase (OPLAH), is responsible for converting 5-oxoproline (5-OP) into glutamate (Glu) [14], which is essential for GSH production [15]. In HF, decreased OPLAH expression disrupts this process, leading to an accumulation of 5-OP and an increase in oxidative stress [16,17]. Studies using mouse models have shown that reduced OPLAH expression results in more severe HF symptoms, while increased OPLAH expression can mitigate disease progression [16].

The accumulation of 5-oxoproline may be particularly relevant in CRS, where oxidative stress affects both the heart and kidneys [7]. Elevated plasma levels of 5-OP have been associated with adverse outcomes in patients with chronic HF [17]. Targeting OPLAH and modulating 5-OP levels could offer a novel therapeutic approach in how we manage both HF and cardiorenal syndrome. Because the kidney is the main site of glutathione turnover, elevated 5-OP in HF patients may reflect an early cardiorenal component of systemic oxidative stress [18,19]. Circulating 5-OP is influenced by kidney function and has been linked to the onset and progression of chronic kidney disease in metabolomics studies [20,21].

Despite evidence linking OPLAH deficiency to HF severity in experimental models, three questions remain unresolved: (1) whether circulating 5-OP predicts adverse clinical outcomes in patients with HF; (2) which circulating molecular signals are most strongly associated with elevated 5-OP; and (3) whether reduced renal OPLAH expression is associated with systemic 5-OP accumulation in a multi-comorbidity setting relevant to cardiorenal disease. To address these questions, we undertook a comprehensive analysis employing three approaches. First, we evaluated the prognostic association of circulating 5-OP with all-cause mortality and heart-failure hospitalization in 823 patients from the BIOSTAT-CHF cohort. Second, we employed stability selection with MCP-penalized regression across 355 circulating proteins to identify robust proteomic correlates of 5-OP. Third, we leveraged a large-animal cardio-kidney-metabolic (CKM) model with renal and cardiac involvement to assess systemic 5-OP levels alongside regional OPLAH expression, emphasizing compartment-specific renal effects.

## 2 Methods

### 2.1 Patient population

A post-hoc analysis was performed utilizing data from the BIOSTAT-CHF (a systems BIOlogy Study to TAilored Treatment in Chronic Heart Failure) index cohort [22,23]. In short, the BIOSTAT-CHF study was a multicenter, multinational, prospective observational investigation involving participants from 11 European countries, with a median follow-up period of 21 months [interquartile range (IQR) 15–27 months]. Eligible participants were adults (≥18 years) presenting with either newly diagnosed or worsening heart failure that was inadequately managed according to established clinical guidelines, characterized by a left ventricular ejection fraction (LVEF) ≤40%, brain natriuretic peptide (BNP) levels >400 pg/mL, or N-terminal pro-brain natriuretic peptide (NT-proBNP) levels >2000 pg/mL.

Patients included in the study were either untreated with angiotensin-converting enzyme inhibitors (ACEi), angiotensin receptor blockers (ARB), and/or β-adrenoreceptor blockers (BB), or were receiving ≤50% of the guideline-recommended target doses, with plans for initiating or uptitrating these therapies. Clinical outcomes were analyzed as specified below (Study Endpoints).

Patients with septicemia, acute myocarditis, hypertrophic obstructive, restrictive, or constrictive cardiomyopathy, or those who had undergone heart transplantation were excluded from the study. Ethical approval for the study protocol was obtained from local and national medical ethics committees (EudraCT 2010-020808-29; R&D Ref Number 2008-CA03; MREC Number 10/S1402/39), and all participants provided written informed consent.

For the current analysis, a subset of 944 patients was selected from the BIOSTAT-CHF index cohort (n = 2516) based on survival status during follow-up, applying a 2:1 ratio of survivors [n = 626 (66%)] to non-survivors [n = 318 (34%)]. Of these individuals, 823 patients (87.2%) had serum available for 5-oxoproline measurements. Baseline 5-oxoproline concentrations were measured in serum and expressed in μM. For descriptive analyses, participants were stratified into tertiles defined using the transformed 5-oxoproline variable applied in regression models (0.5×ln⁡(5‐oxoproline[μM])); however, untransformed concentrations (μM) are reported in Table 1 for interpretability.

**Table 1:** Baseline characteristics across 5-oxoproline tertiles.

Variable	Total Count	Tertile 1	Tertile 2	Tertile 3	P-value
5-oxoproline (μM)	823	27.79 (5.14)	42.38 (4.40)	73.99 (28.95)	<0.0001
Demographics					
Age (years)	71.3 (10.8)	70.8 (11.0)	71.6 (10.7)	71.5 (10.6)	0.60
Male (%)	204 (24.8%)	72 (26.1%)	65 (23.7%)	67 (24.5%)	0.81
Clinical Characteristics					
HF hospitalization in previous year	231 (28.1%)	65 (23.6%)	69 (25.2%)	97 (35.5%)	0.0033
Atrial fibrillation	380 (46.2%)	115 (41.7%)	134 (48.9%)	131 (48.0%)	0.18
Anemia	308 (40.6%)	99 (39.0%)	105 (42.7%)	104 (40.3%)	0.69
Diabetes mellitus	256 (31.1%)	82 (29.7%)	90 (32.8%)	84 (30.8%)	0.72
Renal disease	219 (26.6%)	53 (19.2%)	71 (25.9%)	95 (34.8%)	0.0002
Current alcohol use	316 (38.4%)	89 (32.4%)	70 (25.5%)	61 (22.4%)	0.0272
Physical Examination					
Successful completion of 6MWT (6-Minute Walk Test)	512 (64.9%)	184 (68.9%)	173 (66.8%)	155 (58.9%)	0.0407
6MWT distance	210 (0, 345)	240 (10, 367)	230 (0, 350)	154 (0, 320)	0.0009
Laboratory indices					
NT-proBNP (ng/L)	4064 (2360, 8000)	3301 (1860, 6975)	4206 (2355, 7875)	4741 (2858, 9536)	0.0061
Creatinine (μmol/L)	102 (84, 127)	97.2 (80, 118)	103.3 (83, 131)	109.5 (88.7, 137.9)	<0.0001
eGFR (MDRD) (mL/min/1.73 m^2^)	63.9 (24.9)	67.9 (23.2)	63.9 (25.9)	60.2 (25.1)	0.0012
Renal insufficiency (eGFR <60 mL/min)	389 (47.5%)	106 (38.5%)	135 (49.6%)	148 (54.4%)	0.0007
Urea (mmol/L)	10.6 (7.5, 17.5)	9.4 (6.5, 14.3)	9.9 (7.3, 17.1)	12.5 (8.3, 20.7)	<0.0001
Alkaline phosphatase (μg/L)	85 (66, 116)	77.5 (60.5, 101.5)	83 (66, 122.7)	94 (74, 120.6)	0.0009
Transferrin (mg/dL)	199.99 (66.98)	208.9 (63.4)	199.1 (67.5)	191.7 (69.1)	0.0103
Albumin (g/L)	31.5 (8.5)	33.3 (8.1)	31.4 (8.4)	29.8 (8.8)	<0.0001
CRP (mg/L)	13.9 (5.7, 27.3)	13.8 (5.2, 27.9)	13.4 (6.0, 26.9)	14.8 (6.6, 26.5)	0.96
GDF-15 (ng/mL)	3.7 (1.3)	3.4 (1.2)	3.8 (1.2)	3.96 (1.47)	<0.0001
Medication and Device Therapy					
Use device therapy	208 (25.3%)	52 (18.8%)	65 (23.7%)	91 (33.3%)	0.0004
ACE/ARB (% target dose)	105 (12.8%)	47 (17.0%)	28 (10.2%)	30 (11.0%)	0.0321

### 2.2 Definitions

Estimated glomerular filtration rate (eGFR) was calculated based on the Modification of Diet in Renal Disease (MDRD) equation, and HF with reduced ejection fraction (HFrEF) as a left ventricular ejection fraction < 40%. Serum NT-proBNP concentrations were measured with an electrochemiluminescence immunoassay (Elecsys, Roche Diagnostics), while C-reactive protein (CRP) was measured using competitive immunoassays on a Luminex platform (Alere Inc). Anemia was defined according to the World Health Organization definition hemoglobin level (<12 g/dL in women and <13 g/dL in men).

### 2.3 Study Endpoints

For the present analysis, the primary endpoint was the composite of all-cause mortality or first hospitalization for heart failure, whichever occurred first, censored at 2 years of follow-up. Secondary analyses examined all-cause mortality and heart failure hospitalization as individual components. Endpoints were defined and adjudicated according to the BIOSTAT-CHF study protocol [22,23].

### 2.4 Liquid Chromatography–Mass spectrometry analysis

Serum samples for the measurements were collected at inclusion, either during the index hospitalization or at the outpatient clinic, and were subsequently stored at −80°C.

Quantitative analysis of 5-oxoproline in serum was performed using a Shimadzu HPLC system coupled to an API 4000-2 Triple Quadrupole mass spectrometer, operating in positive Electrospray Ionization (ESI) mode and multiple reaction monitoring (MRM) modes. Chromatographic separation was achieved using a Phenomenex Synergi MAX-RP column (100 × 3 mm, 2.5 μm) with a Phenomenex Synergi MAX-RP C12 guard column (4 × 2 mm), maintained at 40°C. The mobile phase consisted of Eluent A (0.1% formic acid in UP water) and Eluent B (0.1% formic acid in 100% acetonitrile). A gradient elution program was applied as follows: 0.01 min at 3% B, 2.00 min at 3% B, 2.60 min transitioning to MS, 4.00 min at 70% B, 5.00 min returning to 3% B, and 7.00 min stop. The total flow rate over the column was 0.3 mL/min. Mass spectrometry detection was performed using MRM transitions at *m*/*z* 130.0 → 84.1 for 5-oxoproline and *m*/*z* 134.9 → 88.2 for the internal standard (^13^C_5_-labeled 5-oxoproline). The mass spectrometer was configured with the following source and ionization parameters: dwell time of 200 ms for both 5-oxoproline and the internal standard, declustering potential (DP) of 56 for both transitions, entrance potential (EP) of 15 for 5-oxoproline, collision energy (CE) of 16 eV for both analytes, and collision cell exit potential (CXP) of 5 for both transitions. The curtain gas (CAD) was set at 4 units, and the source temperature (TEM) was maintained at 400°C. Serum samples were prepared by diluting 25 μL of serum with 25 μL of internal standard solution (100 μM ^13^C_5_-labeled 5-oxoproline in acetonitrile). The samples were centrifuged at 16,000 × g for 10 min at 4°C. A 10 μL aliquot of the resulting supernatant was transferred into a glass insert vial and diluted with 90 μL of dilution solvent (50% acetonitrile, 50% UP water). An injection volume of 5 μL was used for Liquid Chromatography-Tandem Mass Spectrometry (LC-MS) analysis. Calibration standards were prepared by spiking labeled 5-oxoproline into serum at concentrations ranging from 1 μM to 500 μM, prepared from stock solutions in saline. The calibration curve was constructed by plotting the analyte-to-internal standard peak area ratio versus the known concentration. Quality control (QC) samples were prepared at three concentration levels (low, medium, and high: 7, 30, and 100 μM) and analyzed in triplicate alongside study samples. The system was prepared by purging and equilibrating the HPLC pumps, setting up the correct column, and ensuring all components were operating properly. The autosampler was rinsed, and the ESI probe was positioned correctly before running the acquisition batches.

### 2.5 Biomarker measurements

Serum biomarker concentrations were determined using the proximity extension assay (PEA) technology from OLINK Proteomics (Uppsala, Sweden). This method was employed across four panels, each comprising 92 biomarkers (Cardiovascular II, Cardiovascular III, Immune and Oncology panels), leading to a total of 368 unique biomarkers assessed. Eight biomarkers were excluded from the analysis as more than 10% of their measurements were below the assay's detection limit. The dataset with fewer missing values was used for biomarkers that were measured in duplicate due to overlapping between panels. To maintain consistency in biomarker quantification, NT-proBNP levels from the central laboratory were used rather than the OLINK-derived measurements. Consequently, a final set of 355 biomarkers was included for further statistical analyses.

### 2.6 Biomarker selection and statistical analysis

To identify proteomic associates of circulating 5-oxoproline, we restricted candidate predictors to OLINK proteins (final n = 355) and excluded clinical variables from the penalized feature set. Biomarkers with insufficient variability (fewer than 10 unique values) were removed. Infinite values were set to missing and remaining missing values were imputed using the predictor-specific median, followed by z-standardization of biomarker predictors. We applied stability selection using repeated half-sample subsampling. Across B = 100 iterations, we randomly sampled 50% of participants without replacement and fitted MCP-penalized regression models (ncvreg) along the regularization path [24]. To enforce a fixed model size, we selected the first solution on the path with at least q = 15 non-zero coefficients; if more than q biomarkers were active, we retained the q biomarkers with the largest absolute coefficients. Selection probabilities (π) were computed as the fraction of converged iterations in which each biomarker was selected. For coefficient estimation, we refit an MCP model using the top 20 biomarkers by π, selecting λ via internal cross-validation in cv.ncvreg (default settings) and extracting coefficients at λ_min. Model fit was summarized using the apparent (in-sample) R^2^ and MSE, acknowledging that selection and fitting used the same dataset [[25], [26], [27]]. MCP was chosen because it reduces shrinkage bias for larger coefficients compared with LASSO while retaining variable selection [[26], [27], [28]]. To quantify uncertainty in the apparent model fit, we estimated a 95% bootstrap confidence interval for R^2^ using 500 bootstrap resamples (percentile method). Because biomarker selection and model fitting were performed on the same dataset (with internal cross-validation used only to select λ), these in-sample fit metrics should be interpreted descriptively and may overestimate true predictive performance.

To assess robustness of biomarker selection, we performed two complementary checks. First, we repeated stability selection after residualizing 5-oxoproline with respect to clinically relevant covariates (age, sex, eGFR, NT-proBNP, BMI) using complete cases (n = 795), then reran the same stability selection procedure (B = 100, q = 15). Second, we performed permutation-based null comparisons with N = 1000 outcome permutations (each permutation using B = 100 stability iterations), using both a conservative global test based on the maximum selection probability under the null and a biomarker-specific test focused on TGF-α.

### 2.7 Pathway *enrichment* analysis

To characterize the biological functions of the top 20 biomarkers associated with 5-oxoproline levels, gene set enrichment analysis was performed using Enrichr (https://maayanlab.cloud/Enrichr/) and the STRING database (https://string-db.org/). The following databases were queried: Gene Ontology (Biological Process, Molecular Function, Cellular Component; 2023 release), KEGG (2021 Human), Reactome (2022), WikiPathways (2023 Human), MSigDB Hallmark (2020), and STRING (DISEASES, KEGG, Component). Gene symbols were mapped from UniProt identifiers using the UniProt ID mapping service. Enrichment significance was determined using Fisher's exact test with Benjamini-Hochberg correction for multiple testing [29]. Pathways with adjusted FDR < 0.05 were considered significantly enriched.

### 2.8 Animal experiments

All animal experiments were approved by the Animal Care Committee at the Erasmus University Medical Center (Rotterdam, The Netherlands) and performed in accordance with the “Guiding Principles in the Care and Use of Laboratory Animals” as approved by the Council of the American Physiological Society. Fourteen female (25 ± 1 kg at 2–3 months of age) Yorkshire x Landrace swine were included in which type 2 diabetes mellitus (DM), high-fat diet (HFD), and chronic kidney disease (CKD) were produced, while 12 healthy female swine of similar age served as controls, as previously described [30,31]. Briefly, DM was produced by injection of streptozotocin (50 mg/kg/day i.v. for 3 days, Bio-connect B.V., Huissen, The Netherlands). Ten to fourteen days later, animals were sedated with intramuscular Zoletil (tiletamine/zolazepam; 5 mg/kg), Sedazine (xylazine; 2.25 mg/kg), and atropine (2 mg), and artificially ventilated (O_2_ and N_2_ [1:2]), to which 1–2% (vol/vol) isoflurane was added for anesthesia. CKD was produced by micro-embolization of the global right kidney and the lower pole of the left kidney using 75 mg of polyethylene microspheres (38–42 μm diameter, Cospheric, Santa Barbara, CA, USA) per kidney. One week after CKD induction, a high-fat and high sucrose/fructose diet, supplemented with 20 g NaCl/day, was gradually introduced [30,31].

At six months follow-up, animals in the CKM group displayed significant hyperglycemia, dyslipidemia, renal dysfunction, and systemic inflammation [30,31]. The myocardial tissue showed increased oxidative stress. These pathological changes were associated with increased myocardial collagen deposition, decreased capillary density, and elevated cardiomyocyte stiffness, ultimately resulting in increased left ventricular end-diastolic stiffness, as assessed by pressure-volume loop analysis. Despite these myocardial changes, ejection fraction was maintained, reflecting diastolic dysfunction with preserved systolic function, consistent with the phenotype of heart failure with preserved ejection fraction (HFpEF), as characterized previously [30,31].

### 2.9 OPLAH measurements in swine tissues

Arterial blood samples, and kidney tissue (left/right cortex and medulla) as well as left ventricular (LV) free wall subendocardial tissue were harvested and stored at −80°C. For gene expression analysis, total RNA was isolated from tissue samples using TRIzol reagent according to the manufacturer's protocol. RNA was reverse transcribed to cDNA, and quantitative PCR was performed using primers specific for OPLAH and the housekeeping gene 36b4. Relative gene expression was calculated using the 2^(-ΔΔCt) method. Samples with undetectable OPLAH expression (Ct value = 0) were excluded from the analysis, resulting in final sample sizes of 13 CKM animals for the right kidney cortex and medulla, 12 CKM animals for the medulla and cortex from the non-embolized upper region of the left kidney, and 12 control animals for each region. For Western blot analysis of OPLAH, LV, renal cortex and medulla samples were homogenized and lysed, and protein concentrations were determined to ensure equal loading. Equal amounts of protein (40 μg per lane) were separated by SDS–PAGE and transferred onto PVDF membranes. Prior to transfer, PVDF membranes were pre-wetted in 100% methanol for 1 min. Membranes were blocked in 5% (w/v) non-fat dry milk for 1 h at room temperature and incubated overnight at 4°C with a mouse monoclonal anti-OPLAH antibody (sc-271807, Santa Cruz Biotechnology; 1:100 in 5% milk). α-Tubulin (T5168, Sigma-Aldrich; mouse monoclonal) was used as a loading control (1:8000; overnight at 4°C). Membranes were washed in TBST and incubated with an HRP-conjugated rabbit anti-mouse secondary antibody (P0260, Dako; 1:2000 in 5% milk) for 1 h at room temperature. Bands were visualized using enhanced chemiluminescence and quantified by densitometry. OPLAH signals were normalized to α-tubulin. The same protocol was applied to left ventricular free-wall lysates, with GAPDH (anti-GAPDH antibody; 10R-G109a, Fitzgerald, 1: 25,000 in 5% milk) used as the loading control; LV OPLAH signals were normalized to GAPDH.

### 2.10 5-Oxoproline quantification using LC-MS

To investigate the glutathione cycle's role in the CKM swine model, we quantified serum 5-oxoproline levels using a previously validated LC-MS method. Serum samples were prepared by adding 25 μL of extraction solution containing 100 μM ^13^C_5_-5-oxoproline in acetonitrile. After centrifugation (16,000 × g, 10 min, 4°C), 10 μL of supernatant was diluted with 90 μL of dilution solvent (50:50 acetonitrile:water). Analysis was performed using a Shimadzu HPLC system coupled to an API 4000-2 Triple Quadrupole mass spectrometer in positive ESI mode. Separation was achieved on a Phenomenex Synergi MAX-RP column (100 × 3 mm, 2.5 μm) at 40°C, using a mobile phase of 0.1% formic acid in water (A) and acetonitrile (B) at 0.3 mL/min. MRM transitions were monitored at *m*/*z* 130.0 → 84.1 for 5-oxoproline and *m*/*z* 134.9 → 88.2 for ^13^C_5_-labeled internal standard, with the mass spectrometer operating at 400°C. The injection volume was 5 μL.

### 2.11 Statistical analysis

All statistical analyses were performed using STATA v.18 SE and R Version 4.5.2. For the BIOSTAT-CHF cohort, patients were divided into tertiles based on 5-oxoproline concentration. Variables with normal distribution were reported as mean (standard deviation), continuous variables with non-normal distribution as median (interquartile range), and categorical variables as number (percentage). Baseline characteristics across 5-oxoproline tertiles were analyzed using one-way analysis of variance (ANOVA) for continuous variables with normal distribution, the Kruskal–Wallis test for continuous variables with skewed data, and the Chi-squared test for categorical variables.

Survival analyses were performed using Kaplan-Meier curves and Cox proportional hazards regression. Hazard ratios were calculated for the association between 5-oxoproline levels (both as tertiles and as a continuous variable) and clinical outcomes, including all-cause mortality and heart failure hospitalization. Models were adjusted using the BIOSTAT-CHF risk model variables.

For the animal studies, data were first screened for outliers using the Interquartile Range (IQR) method; values falling below Q1 – 1.5×IQR or above Q3 + 1.5×IQR were considered outliers and excluded to ensure data robustness. The final sample sizes after outlier exclusion are specified in each figure. The normality of data distribution was assessed using the Shapiro-Wilk test.

Comparisons between two independent groups (CKM vs. Control) were performed using the Unpaired Student's t-test for normally distributed data with equal variances. For data that did not follow a normal distribution or exhibited unequal variances (including serum 5-oxoproline, glutamate, and specific renal OPLAH datasets), the Exact Mann-Whitney *U* test or Welch's *t*-test was utilized as appropriate.

For protein expression comparisons, band intensities were normalized to loading controls indicated for each tissue (GAPDH or Tubulin). All statistical analyses were performed using Python (scipy.stats) and GraphPad Prism. A two-tailed p-value < 0.05 was considered statistically significant.

## 3 Results

### 3.1 Baseline clinical characteristics

In the BIOSTAT-CHF index cohort, serum 5-oxoproline levels were measured in a subset of 823 patients (as summarized in Table 1). The cohort was stratified into tertiles of transformed 5-oxoproline (0.5×ln⁡[μM]). Untransformed 5-oxoproline concentrations rose progressively across tertiles: T1 27.8 ± 5.1 μM, T2 42.4 ± 4.4 μM, and T3 74.0 ± 29.0 μM (p < 0.0001). Patients in the highest tertile exhibited a markedly higher burden of renal disease (34.8% vs 19.2% in T1, p = 0.0002), evidenced by impaired renal function (mean eGFR falling from 67.9 ± 23.2 in T1 to 60.2 ± 25.1 mL/min/1.73 m^2^ in T3; p = 0.0012 for the across-tertile comparison) and a higher prevalence of renal insufficiency (eGFR < 60 mL/min; 54.4% vs 38.5%, p = 0.0007). Consistent with kidney dysfunction, laboratory markers in the highest tertile showed higher levels of creatinine (109.5 vs 97.2 μmol/L, p < 0.0001), urea (12.5 vs 9.4 mmol/L, p < 0.0001), growth differentiation factor 15 (GDF-15) (3.96 vs 3.4 ng/mL, p < 0.0001) and alkaline phosphatase (94 vs 77.5 μg/L, p = 0.0009). While C-reactive protein levels did not differ significantly (14.8 vs 13.8 mg/L, p = 0.96), other markers suggested heightened inflammatory activity**.**

These patients also demonstrated more frequent HF hospitalizations in the previous year (35.5% vs 23.6% in T1, p = 0.0033) and greater device therapy utilization (33.3% vs 18.8% in T1, p = 0.0004). Their reduced exercise capacity was notable in the highest tertile, evidenced by lower 6-min walk test completion rates (58.9% vs 68.9% in T1, p = 0.0407) and shorter walking distances (154 m vs 240 m in T1, p = 0.0009). Finally, NT-proBNP levels were significantly higher in patients with highest 5-oxoproline levels (4741 vs 3301 ng/L in T1, p = 0.0061), along with significantly lower albumin (29.8 vs 33.3 g/L, p < 0.0001) and transferrin levels (191.7 vs 208.9 mg/dL, p = 0.0103).

High 5-oxoproline levels associate with higher all-cause mortality and hospitalization for heart *failure*.

**Fig. 1:**
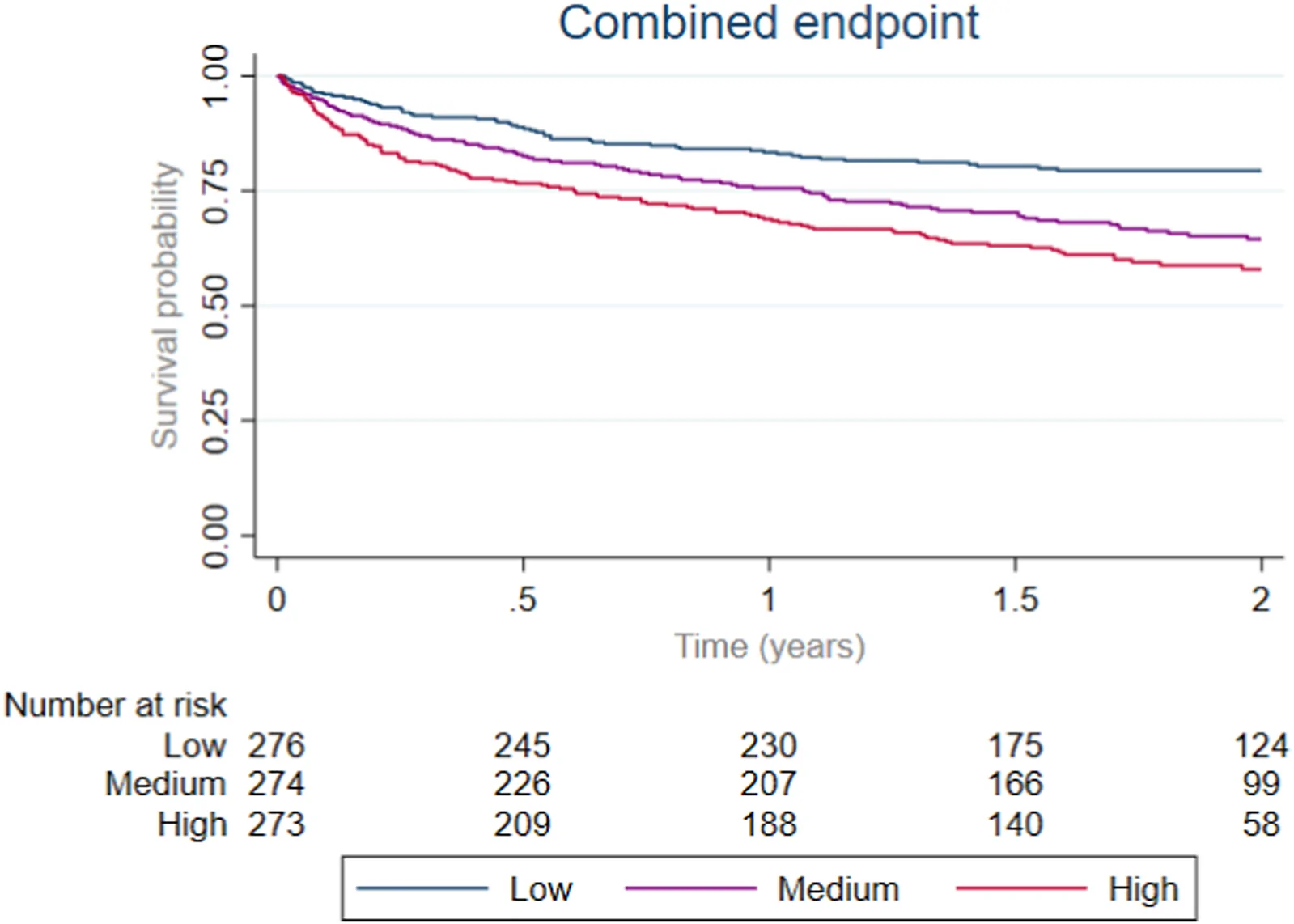
**Kaplan–Meier Curves for combined endpoint by 5-Oxoproline Tertiles** Patients were stratified into tertiles based on serum 5-oxoproline levels: Low (Tertile 1, n = 276), Medium (Tertile 2, n = 274), and High (Tertile 3, n = 273). After adjustment for BIOSTAT-CHF risk model variables, patients in the highest tertile showed significantly increased risk compared with the lowest tertile (HR 1.65, 95% CI 1.19–2.28, p = 0.003). The second tertile also demonstrated elevated risk (HR 1.45, 95% CI 1.04–2.03, p = 0.028). Number at risk is shown below the graph. Abbreviations: HR, hazard ratio; CI, confidence interval.

**Fig. 2:**
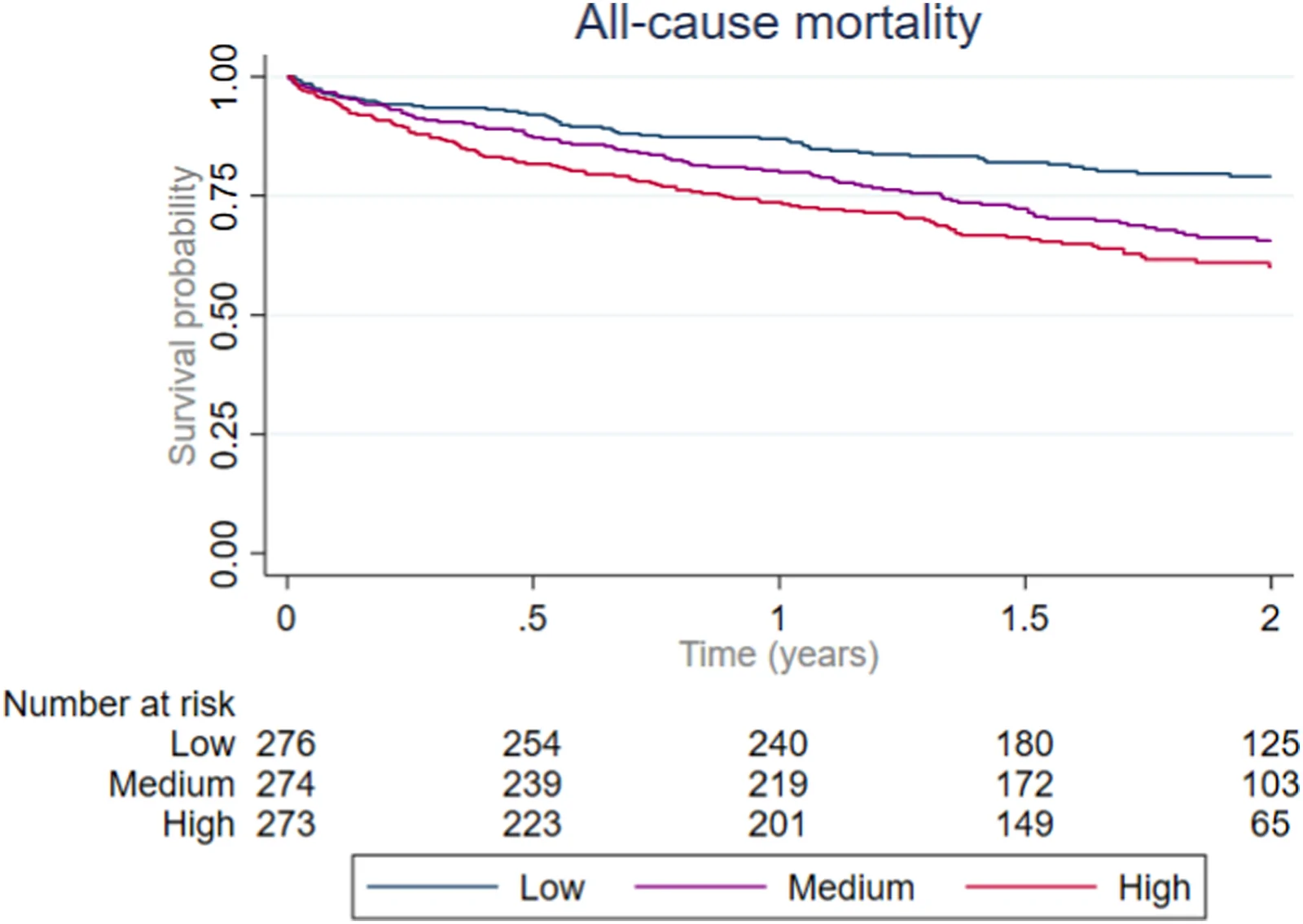
**Kaplan–Meier Curves for All-Cause Mortality by 5-Oxoproline Tertiles** Patients were stratified into tertiles based on serum 5-oxoproline levels: Low (Tertile 1, n = 276), Medium (Tertile 2, n = 274), and High (Tertile 3, n = 273). After adjustment for BIOSTAT-CHF risk model variables, patients in the highest tertile showed significantly increased mortality risk compared with the lowest tertile (HR 1.55, 95% CI 1.11–2.17, p = 0.010). The second tertile also demonstrated elevated risk (HR 1.42, 95% CI 1.01–1.99, p = 0.044). Number at risk is shown below the graph. Abbreviations: HR, hazard ratio; CI, confidence interval.

During a median follow-up of 21 months (IQR 15–27), elevated 5-oxoproline levels demonstrated strong associations with adverse outcomes. After adjusting for the BIOSTAT-CHF risk model, patients in the highest tertile faced a 65% increased risk for the combined endpoint of death or heart failure hospitalization (HR 1.65, 95% CI 1.19–2.28, p = 0.003; Fig. 1), a 55% increased risk for all-cause mortality (HR 1.55, 95% CI 1.11–2.17, p = 0.010; Fig. 2), and an 81% increased risk for heart failure hospitalization alone (HR 1.81, 95% CI 1.08–3.03, p = 0.024). Analysis of 5-oxoproline as a continuous variable yielded consistent findings: each 1-SD increase in the transformed 5-oxoproline metric (0.5×ln(5‐oxoproline[μM])) was associated with higher risk across endpoints (Table 2). Given the link between 5-OP biology, glutathione depletion, and renal oxidative stress, we examined whether associations differed by CKD status (Table S2). After full adjustment, each SD increase in 5-OP raised the composite 2-year risk by 25% in CKD (HR 1.25, 95% CI 1.05–1.48, p = 0.012) and by 17% in those without CKD (HR 1.17, 95% CI 0.96–1.42, p = 0.12). The formal CKD × 5-OP interaction was not statistically significant (p = 0.63), suggesting a consistent prognostic value independent of renal strata (Table S2).

**Table 2:** Association between serum 5-oxoproline levels and clinical outcomes in heart failure patients.

Outcome and Model	Events/N	Hazard Ratio (95% CI)	P-value
Combined Endpoint	258/823		
5-oxoproline (tertiles)^a^			
- Second tertile		1.45 (1.04-2.03)	0.028
- Third tertile		1.65 (1.19-2.28)	0.003
5-oxoproline (continuous)^b^		1.27 (1.05-1.53)	0.015
All-Cause Mortality	246/823		
5-oxoproline (tertiles)^a^			
- Second tertile		1.42 (1.01-1.99)	0.044
- Third tertile		1.55 (1.11-2.17)	0.010
5-oxoproline (continuous)^b^		1.24 (1.02-1.51)	0.028
HF Hospitalization	107/823		
5-oxoproline (tertiles)^a^			
- Second tertile		1.54 (0.91-2.61)	0.110
- Third tertile		1.81 (1.08-3.03)	0.024
5-oxoproline (continuous)^b^		1.40 (1.05-1.86)	0.022

a All models adjusted for BIOSTAT-CHF risk model variables [22].

b Hazard ratios for continuous measurements represent risk per 1-SD increase in log-transformed 5-oxoproline levels. Abbreviations: CI, confidence interval; HF, heart failure; N, number of patients; SD, standard deviation.

### 3.2 TGF-α presents as a robust molecular correlate of circulating 5-oxoproline levels

To delineate the proteomic landscape linked to 5-OP levels, we analyzed 355 circulating proteins using the OLINK platform and applied stability selection with MCP, focusing initially on proteomic predictors alone. Demographic and clinical variables were omitted from this initial step to prioritize the identification of proteomic predictors; potential confounding by key clinical covariates was explored in a sensitivity analysis by residualizing 5-OP for age, sex, eGFR, NT-proBNP, and BMI prior to selection. The stability selection procedure used B = 100 half-sample subsampling iterations (all iterations completed without numerical issues).

Transforming growth factor-α (TGF-α) emerged as the most robust biomarker associated with 5-OP levels (standardized coefficient = 0.211, selection probability π = 0.70), exceeding the second-ranked biomarker (MMP7, π = 0.55) and meeting the pre-specified robustness threshold (π ≥ 0.70). To validate that this selection probability was not an artifact of chance, we performed permutation testing with N = 1000 outcome permutations using identical stability-selection parameters (B = 100, q = 15). Under the TGF-α–specific null distribution, none of the 1000 permutations reached the observed π value. This corresponds to an empirical p-value of 0.001, calculated using the +1 correction ((0 + 1)/(1000 + 1); Fig. S1). As expected for a single marker, the more conservative global test, which requires TGF-α to exceed the maximum selection probability across all 355 proteins, was not significant (p = 0.183, Fig. S2), reflecting its stringent panel-wide threshold rather than a weak signal.

**Fig. 3:**
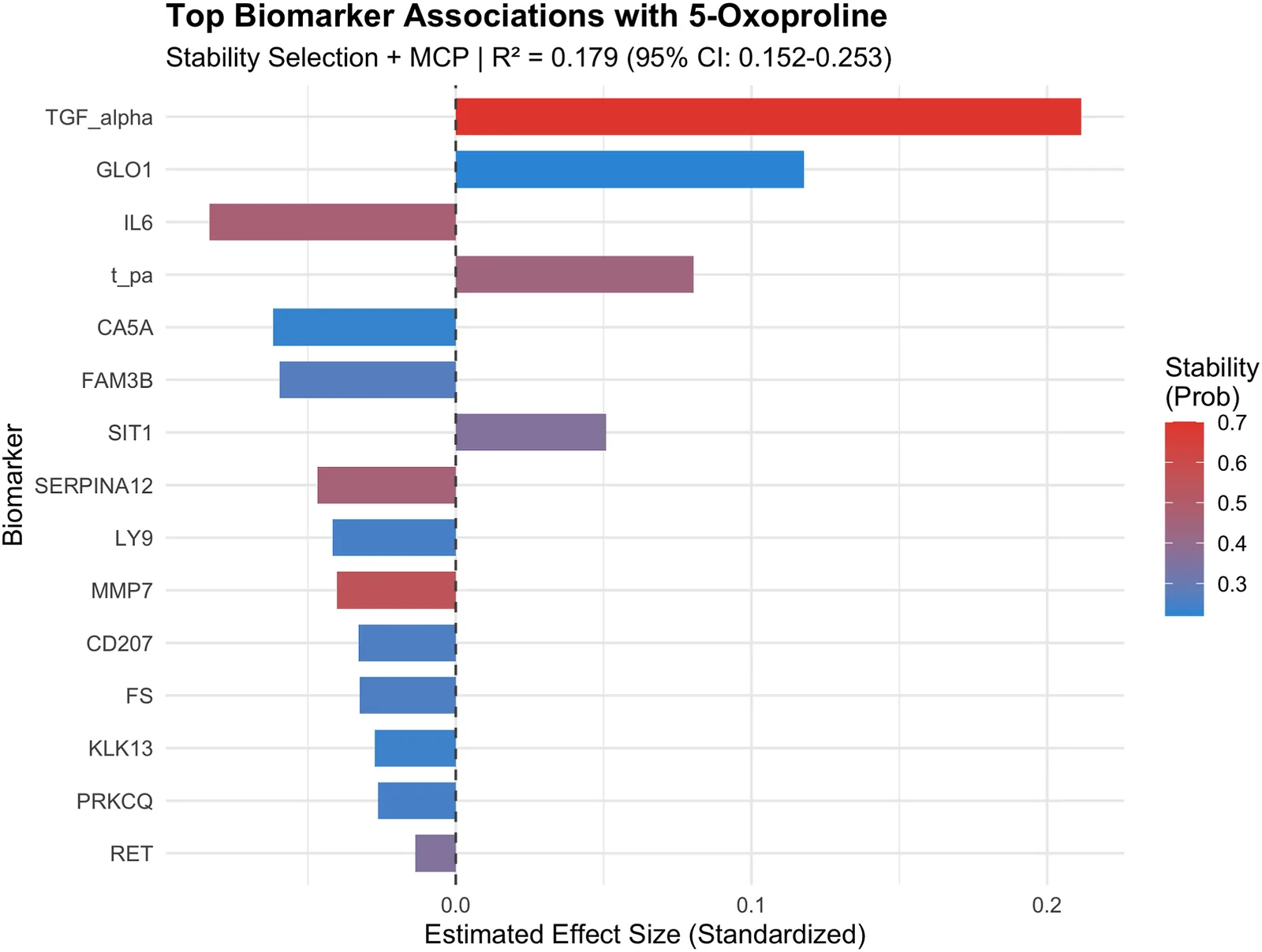
Top 15 of the 20 highest-ranked biomarkers associated with circulating 5-oxoproline levels. Bar plot showing standardized effect sizes (x-axis) and stability selection probabilities (π; color scale) for the top 15 biomarkers ranked by π, identified using stability selection with MCP-penalized regression. TGF-α showed the highest selection probability (π = 0.70) and was the only biomarker meeting the pre-specified robustness threshold (π ≥ 0.70). Positive coefficients indicate biomarkers that increase with higher 5-oxoproline; negative coefficients indicate inverse associations. The MSE of 0.334 and an R^2^ of 0.179 (95% bootstrap CI: 0.152–0.253).

Matrix metalloproteinase-7 (MMP7) was the second most frequently selected biomarker (π = 0.55), exhibiting an inverse relationship with 5-OP (coefficient = −0.040). Other biomarkers demonstrating moderate selection probabilities included interleukin-6 (IL-6, π = 0.47), tissue plasminogen activator (t-PA, π = 0.44), and RET proto-oncogene (π = 0.36). Glyoxalase I (GLO1, π = 0.22) also showed detectable selection frequency, hinting at the potential involvement of carbonyl-stress–related pathways within the 5-OP-associated proteomic signature. The top 20 biomarkers are summarized in Table S4, with the top 15 visualized in Fig. 3.

To determine whether TGF-α represented an isolated signal or part of a broader tissue-remodeling response, we performed a targeted post hoc re-analysis of circulating tissue remodeling/renal injury-related proteins and redox/stress-related proteins across the full 355-protein panel, applying Benjamini-Hochberg (BH) correction across all proteins. Of ten remodeling/renal injury-related proteins, seven remained positively associated with 5-OP after full-panel BH correction: DCN (r = 0.127, BH p = 0.003), VIM (r = 0.124, BH p = 0.004), TGFBR2 (r = 0.116, BH p = 0.007), MMP-9 (r = 0.112, BH p = 0.010), TGM2 (r = 0.104, BH p = 0.017), KRT19 (r = 0.094, BH p = 0.032), and OLR1/LOX-1 (r = 0.088, BH p = 0.044); nine of ten were directionally positive. In contrast, several canonical matrix/fibrosis markers on the same panel (collagen I, MMP-2, PAI-1, and osteopontin) were not associated with 5-OP. Among redox/stress-related proteins, GLO1 (r = 0.093, BH p = 0.034) and EGLN1 (r = 0.098, BH p = 0.024) were individually associated, without a clear group-level redox pattern. This targeted analysis is exploratory and hypothesis-generating; it indicates that the TGF-α association is accompanied by several individually significant, biologically related remodeling proteins rather than representing an isolated finding (Fig. S3, Table S6).

To assess robustness to clinical confounding, we repeated stability selection after residualizing 5-oxoproline for age, sex, eGFR, NT-proBNP, and BMI. TGF-α maintained the highest selection probability after residualization (adjusted π = 0.60; reflecting a 14% decrease from unadjusted). The standardized coefficient increased moderately after adjustment (0.211 → 0.249), suggesting that the TGF-α–5-OP association captures biological insight independent of renal filtration and cardiac stress markers alone. The top-ranked biomarkers showed stable selection probabilities after adjustment (Table S4 and Table S3 for all 355 proteins). The two null distributions are compared in Fig. S2.

The MCP model incorporating the top 20 biomarkers produced an MSE of 0.334 and an R^2^ of 0.179 (95% bootstrap CI: 0.152–0.253). It is important to note that these metrics may reflect optimistic estimates of predictive performance, as selection and fitting occurred on the same dataset. External validation would be required for an unbiased performance assessment.

### 3.3 Pathway enrichment analysis

**Fig. 4:**
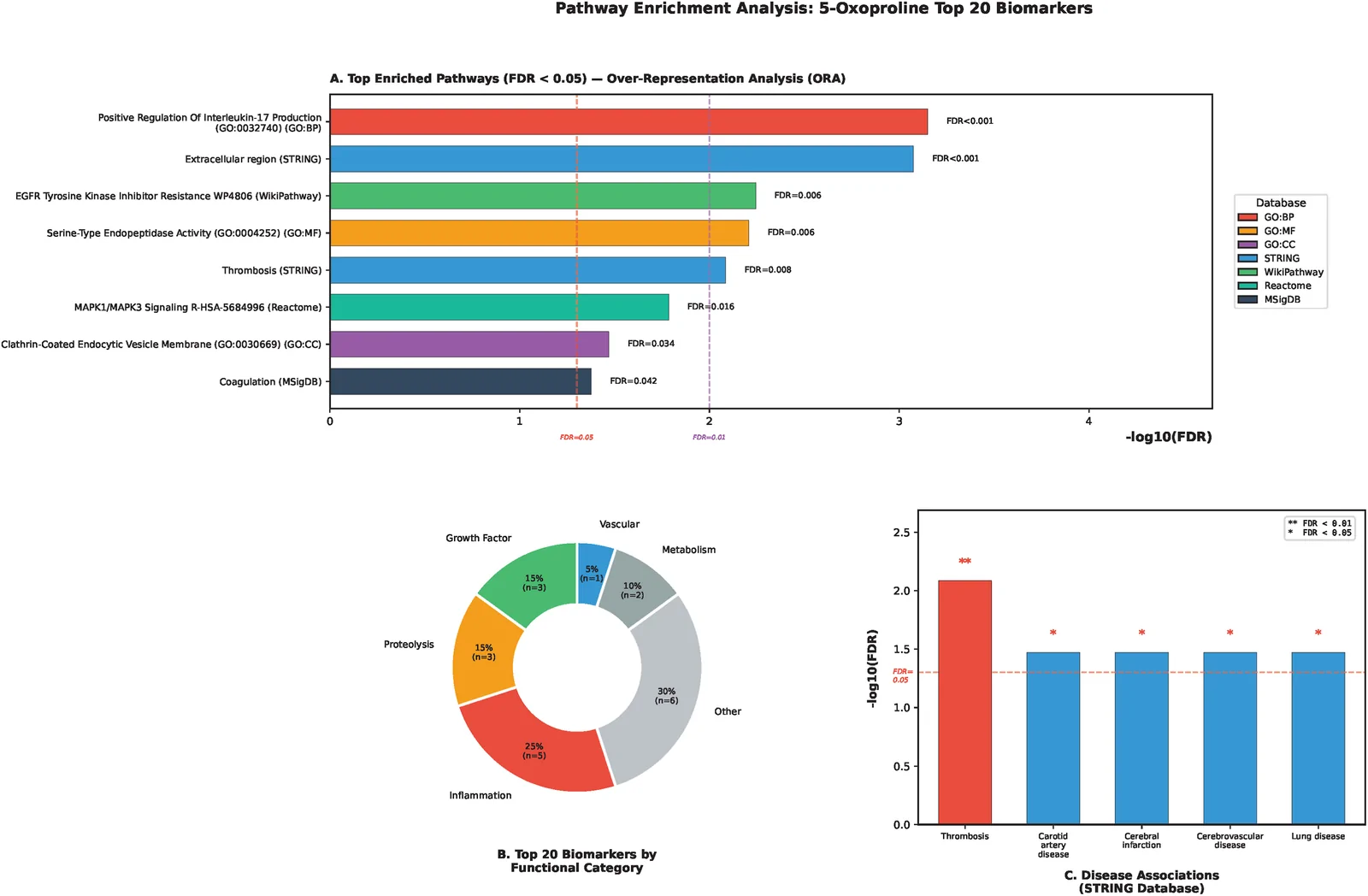
**Pathway enrichment analysis of top 20 biomarkers associated with 5-oxoproline**. (A) Top enriched pathways across multiple databases showing significant enrichment for inflammatory processes (Th17/IL-17 differentiation), growth factor signaling (EGFR/MAPK), and thrombosis-related pathways. (B) Distribution of biomarkers by functional category. (C) Disease associations from STRING database, with thrombosis showing the strongest enrichment (FDR = 8.2 × 10^−3^). All p-values are FDR-adjusted (Benjamini-Hochberg correction). Dashed lines indicate significance thresholds (FDR = 0.05 and FDR = 0.01).

To assess the biological significance of the top 20 biomarkers associated with 5-oxoproline, we performed a gene set enrichment analysis across multiple databases (Gene Ontology, KEGG, Reactome, WikiPathways). TGF-α, as the primary ligand for epidermal growth factor receptor (EGFR), was central to the enrichment of EGFR tyrosine kinase signaling (WikiPathway FDR = 5.71 × 10^−3^) and downstream MAPK1/MAPK3 signaling cascades (Reactome FDR = 1.64 × 10^−2^). These enrichment results are summarized in Fig. 4 and Table S5.

Inflammatory pathways were notably enriched, particularly Th17 cell differentiation and positive regulation of interleukin-17 production (Gene Ontology FDR = 7.07 × 10^−4^), driven by IL6, PRKCQ, and LY9. Disease association analysis highlighted a strong link to thrombosis (FDR = 8.2 × 10^−3^), cerebral infarction (FDR = 3.36 × 10^−2^), and carotid artery disease (FDR = 3.36 × 10^−2^), with key contributions from tissue plasminogen activator (PLAT), P-selectin (SELP), and IL-6. These findings suggest that elevated 5-OP, through its associated biomarker network, aligns with broader systemic oxidative stress involving growth factor signaling, inflammatory responses, and vascular function (Table S5).

### 3.4 Systemic 5-oxoproline accumulation is more consistent with a renal than a cardiac contribution

**Fig. 5:**
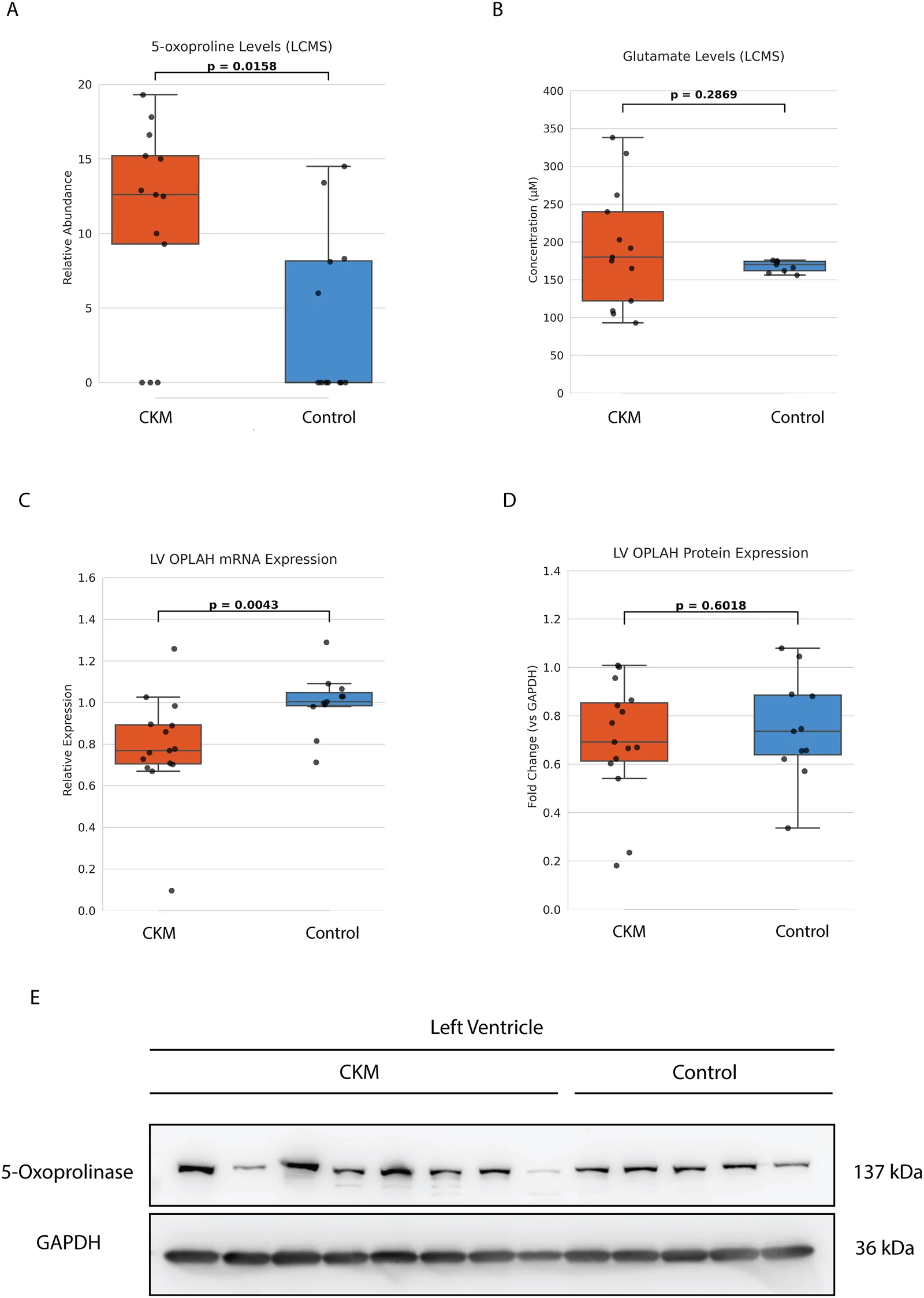
**Systemic 5-oxoproline/Glutamate levels and Left Ventricular OPLAH expression. (A)** Serum 5-oxoproline levels measured by LC-MS were significantly higher in the CKM group (p = 0.0158, Exact Mann-Whitney *U* test). **(B)** Serum Glutamate levels showed no significant difference between groups (p = 0.2869, Welch's *t*-test), indicating a specific defect in 5-oxoproline conversion rather than general cycle metabolite accumulation. **(C)***OPLAH* mRNA expression was significantly downregulated in the Left Ventricle of CKM animals (p = 0.0043). **(D)** OPLAH protein expression in the Left Ventricle showed no significant difference between groups (p = 0.6018). **(E)** Representative Western Blot images for the Left Ventricle (GAPDH used as loading control). Data are presented as box plots with individual data points; outliers were excluded using the IQR method**.**

To examine the metabolic basis of cardiorenal stress, we utilized a large-animal CKM model. LC–MS analysis revealed significantly elevated serum 5-oxoproline levels in CKM animals compared with controls (p = 0.0158; Fig. 5A). Importantly, systemic glutamate, the direct product of 5-OP conversion, remained unchanged (p = 0.2869; Fig. 5B). This dissociation is indicative of a selective perturbation in 5-oxoproline conversion/handling rather than a broad disruption of γ-glutamyl cycle output.

We next assessed whether cardiac OPLAH changes mirrored the systemic elevation. While LV OPLAH mRNA expression was significantly downregulated in CKM animals (p = 0.0043; Fig. 5C), this transcriptional change was not accompanied by a reduction in LV OPLAH protein abundance, which remained comparable to controls (p = 0.6018; Fig. 5D). Together, these findings suggest that reduced LV OPLAH transcription is uncoupled from protein abundance at the sampled timepoint, rendering the heart as an unlikely primary driver of the systemic 5-oxoproline elevation observed in CKM animals.

### 3.5 Compartment-specific loss of renal OPLAH

**Fig. 6:**
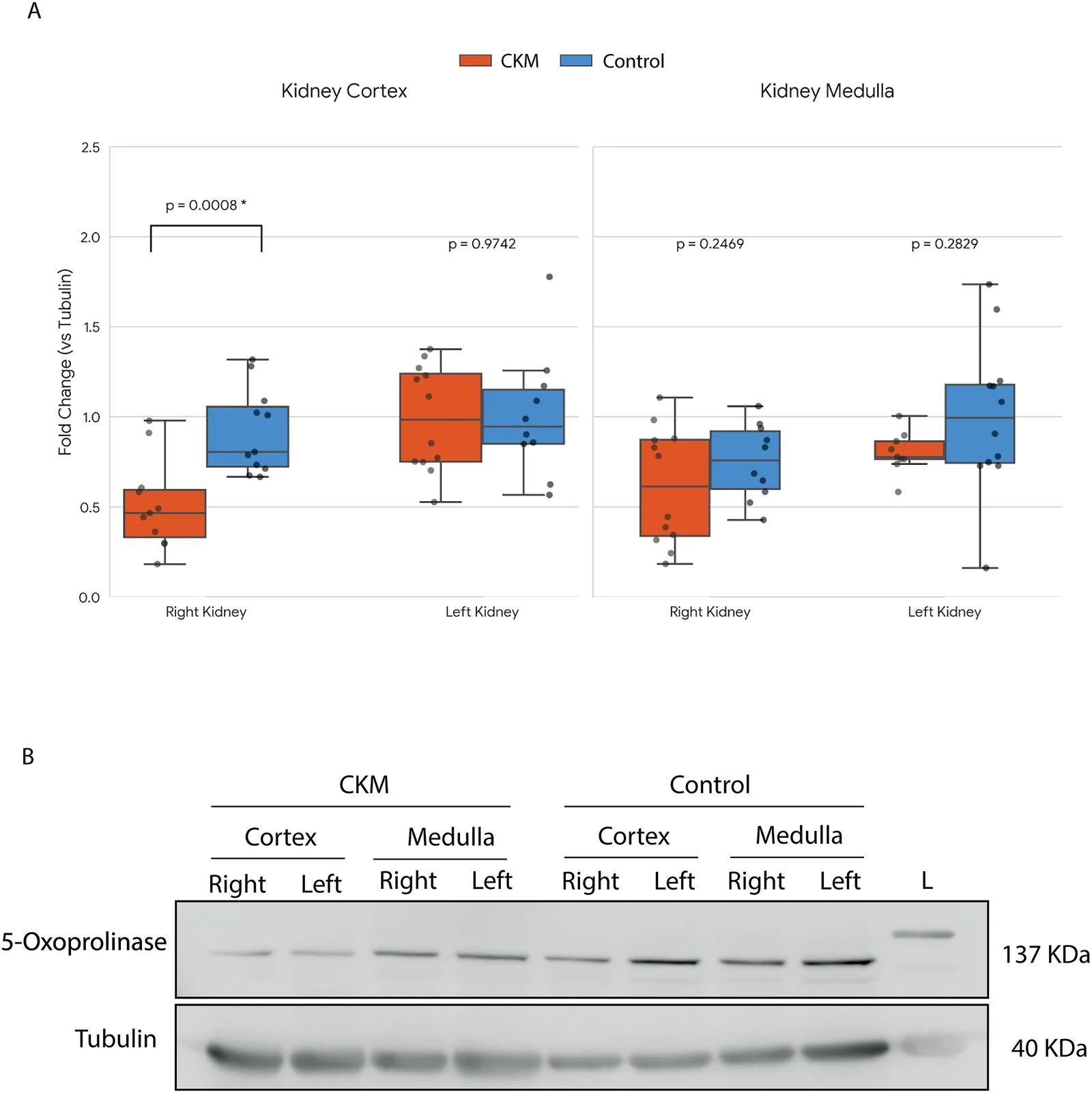
**OPLAH protein expression in preserved vs. embolized kidney tissues. (A)** OPLAH protein expression in the Kidney Cortex and Medulla. The embolized Right Kidney Cortex of CKM swine shows a significant reduction in OPLAH levels compared to Controls (p = 0.0008), consistent with regionally impaired cortical tissue in the embolized kidney. In contrast, the Left Kidney Cortex (preserved upper pole) maintains OPLAH expression levels comparable to Controls (p = 0.9742), indicating metabolic preservation in the functional tissue. No significant differences were observed in the medulla. **(B)** Representative Western Blot images (Tubulin used as loading control). Data represent fold change relative to Control means. Statistical significance was determined using the Exact Mann-Whitney *U* test. Outliers were identified and removed using the IQR method.

Given the kidney's substantial role in glutathione turnover, we examined renal OPLAH expression by compartment. Western blot analysis revealed distinct compartment-specific differences (Fig. 6). Within the embolized right kidney cortex, OPLAH protein expression was significantly reduced in CKM animals compared with Controls (p = 0.0008; Fig. 6A). In contrast, the preserved cortex of the left kidney (upper pole) maintained OPLAH expression levels comparable to Controls (p = 0.9742), despite the systemic milieu. No significant differences were observed in the renal medulla of either kidney (p > 0.05). These findings support the concept that systemic 5-OP accumulation may stem from the selective loss of OPLAH in the damaged renal cortex.

## 4 Discussion

Our results indicate that elevated circulating 5-OP levels correlate with deteriorating renal function and a more severe HF clinical profile. Patients with higher 5-OP levels exhibited increased risks of all-cause mortality and heart failure hospitalization, associations that persisted even after adjusting for established risk factors. While point estimates were numerically higher in patients with CKD (HR 1.25) than without (HR 1.17), the formal interaction test was not significant (p = 0.63), indicating consistent prognostic associations across renal strata. These associations remained significant after adjustment for traditional prognostic markers, suggesting potential clinical value in risk stratification of patients with HF and renal disease. Our finding parallels recent research demonstrating that 5-OP could serve as a reliable marker of oxidative stress in HF [16,17] and may reflect a disrupted gamma-glutamyl cycle in cardiorenal dysfunction. The functional importance of OPLAH in mediating 5-OP metabolism has been demonstrated in experimental models of cardiac injury, where 5-OP requires functional OPLAH to exert cardioprotective effects through conversion to glutamate and subsequent GSH synthesis [32]. Consequently, OPLAH deficiency leads to 5-OP accumulation and cardiac dysfunction [16,17], supporting the concept that elevated circulating 5-OP in our HF patients might reflect impaired metabolism rather than functional protection, thereby serving as a biomarker of disrupted gamma-glutamyl cycle function.

**Fig. 7:**
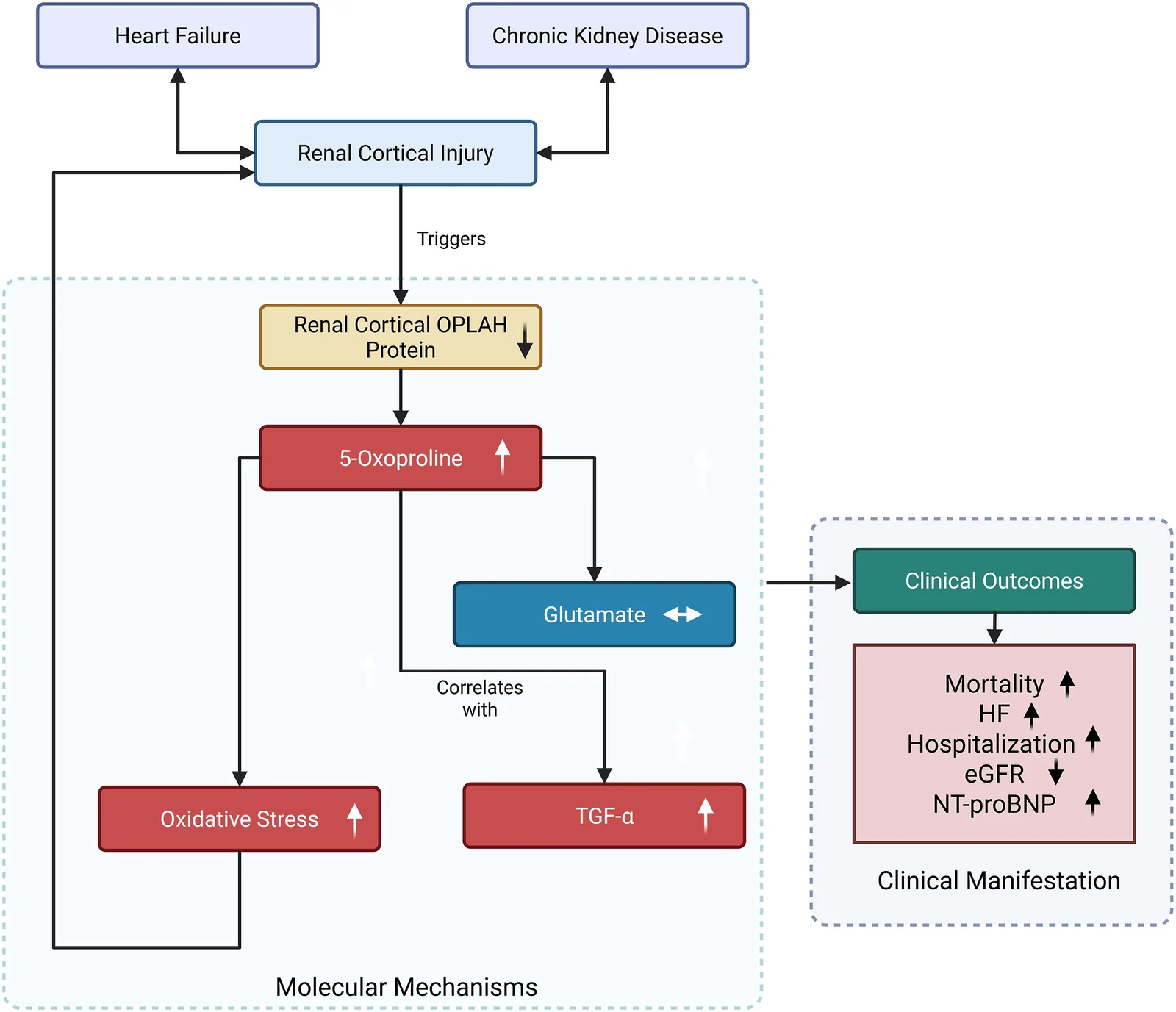
**Integrated pathophysiological model linking renal cortical OPLAH dysfunction to cardiorenal syndrome.** Heart failure and chronic kidney disease promote renal cortical injury, which in turn leads to a selective reduction of renal cortical OPLAH protein abundance (consistent with reduced enzymatic capacity) and increased circulating 5-oxoproline. Impaired OPLAH activity causes accumulation of 5-oxoproline while systemic glutamate levels remain preserved, indicating a specific bottleneck in the gamma-glutamyl cycle rather than a global depletion of its substrates. Increased 5-oxoproline is associated with higher TGF-α levels and enhanced oxidative stress, potentially reinforcing renal and cardiac damage in a feed-forward loop. Clinically, this metabolic signature is linked to adverse outcomes, including increased mortality, heart failure hospitalization, lower eGFR, and higher NT-proBNP. Altogether, the model supports the injured renal cortex as a likely contributor to systemic 5-oxoproline elevation in this setting.

The proteomic analysis identified TGF-α as the most robust molecular correlate of circulating 5-oxoproline levels (coefficient = 0.211, selection probability = 0.70). TGF-α was notably the sole biomarker to surpass the stringent threshold for robust selection among the 355 circulating proteins evaluated, underscoring the specificity of this association. TGF-α is a multifaceted growth factor that drives inflammation, fibrosis, and maladaptive repair in cardiorenal pathology. In the kidney, TGF-α mediated EGFR activation promotes diabetic nephropathy and renal fibrosis [33,34]. Concurrently, in cardiac tissue, TGF-α acts as a critical downstream mediator of Angiotensin II, driving cardiomyocyte hypertrophy and remodeling via the same EGFR signaling axis [35]. Urinary TGF-α has also been incorporated into a multi-biomarker model that predicts CKD progression [36]; its correlation with 5-oxoproline in our data may indicate a common oxidative stress pathway. Additionally, secondary biomarkers with moderate selection probabilities included MMP7 (π = 0.55), involved in extracellular matrix remodeling, IL-6 (π = 0.47), a key inflammatory cytokine, and t-PA (π = 0.44), linked to fibrinolysis and thrombotic risk. Prior studies demonstrate that intrarenal TGF-α mRNA expression and both urine and serum TGF-α levels increase in diabetic kidney disease, and that TGF-α neutralization attenuates renal injury in diabetic mice, particularly when combined with renin–angiotensin system inhibition [33]. Similarly, TGF-α is identified as a key mediator of genetic susceptibility to CKD in various experimental models [37]. The convergence of elevated 5-OP (indicating oxidative stress and impaired gamma-glutamyl cycle function) with increased TGF-α (a well-known driver of renal fibrosis and dysfunction) suggests a potential mechanistic pathway in the pathophysiology of CRS (Fig. 7). That several individually significant, biologically related remodeling proteins accompanied TGF-α argues against an isolated or anecdotal signal, while the cellular localization and directionality of this association remain to be defined.

Furthermore, the enrichment for Th17 cell differentiation and IL-17 signaling provides a novel link between 5-oxoproline and systemic inflammatory activation. IL-6, among our top-ranked biomarkers, is a regulator of Th17 differentiation and has been implicated in the pathogenesis of HF through mechanisms including endothelial dysfunction and tissue remodeling [38,39]. Emerging evidence suggests that patients with heart failure exhibit higher frequencies of proinflammatory Th17 cells and a Th17/Treg imbalance, features that correlate with disease severity [40,41].

In the large-animal CKM model, we observed an increase in systemic 5-oxoproline levels, reflecting the direction of our clinical findings. When focusing on the kidneys, we identified regional differences in OPLAH mRNA and protein expression following selective embolization. Compared with healthy controls, the completely (diffusely) embolized right kidney showed significantly reduced OPLAH expression, most prominently in the cortex. In contrast, the preserved (non-embolized) cortical regions of the partially (regionally) embolized left kidney maintained OPLAH expression levels comparable to controls. The regional differences in OPLAH expression paralleled the extent of tissue injury induced by embolization, supporting an association between cortical damage and reduced OPLAH protein abundance. These differences co-occurred with changes in systemic 5-oxoproline in these animals. Published characterization of this same multi-comorbidity CKM model reports embolization-induced hypertension, reduced glomerular filtration, renal tubulointerstitial injury, and cardiac oxidative stress, providing hemodynamic and tissue context for the present findings [30,31].

Our experimental findings provide additional context for systemic 5-oxoproline accumulation in cardiorenal stress. The observation that circulating glutamate levels remained unchanged despite elevated 5-oxoproline is more consistent with a selective metabolic bottleneck than with a global disruption of downstream circulating γ-glutamyl cycle products. Although cardiac OPLAH mRNA expression was reduced, myocardial OPLAH protein abundance was preserved, suggesting that transcriptional changes did not translate into measurable changes in protein levels at the sampled timepoint. In contrast, the injured renal cortex demonstrated a clear and regionally consistent loss of OPLAH protein, closely mirroring the underlying pattern of tissue damage. These findings collectively suggest that loss of renal cortical OPLAH protein (and thus potentially reduced enzymatic capacity), rather than myocardial changes in OPLAH protein abundance, may contribute more strongly to systemic 5-oxoproline accumulation, reinforcing the kidney's prominent role in glutathione turnover and redox homeostasis.

The regional specificity of OPLAH reduction aligns with the differential vulnerability of nephron segments to injury, as the renal cortex contains critical filtration structures more susceptible to ischemic damage [42]. This is in line with evidence demonstrating the kidney's capacity to maintain glutathione metabolism even during significant oxidative stress [43], although this capacity may become regionally compromised with injury. The maintenance of OPLAH expression in preserved kidney tissue suggests that protecting remaining functional nephrons may be important for metabolic homeostasis in the context of partial kidney damage. This nephron protection concept is supported by broad evidence that preserved nephron function contributes to renal homeostasis across multiple forms of kidney disease [44].

From a clinical perspective, these findings suggest potential applications for risk stratification in heart failure. The association between 5-oxoproline levels and adverse outcomes supports its utility as a circulating biomarker reflecting cardiorenal stress biology, particularly for identifying patients at higher risk of disease progression [45]. Furthermore, the observation that preserved kidney tissue maintains OPLAH expression provides a metabolic rationale for the clinical paradigm of nephron protection. This suggests that therapeutic strategies aimed at preserving functional renal mass are critical not only for filtration but also for maintaining systemic glutathione homeostasis and redox balance in cardiorenal syndrome.

### 4.1 Study limitations

Our findings should be interpreted in light of several limitations. First, the observational design of the clinical analyses supports association rather than causation between circulating 5-oxoproline, TGFα, and outcomes. Although we adjusted for established prognostic factors, unmeasured or residual confounding cannot be fully excluded. Second, the CKM model provides translational context and tissue-level resolution, but it cannot recapitulate all aspects of human cardiorenal syndrome, particularly the long-term chronicity and heterogeneity of disease evolution; more generally, animal models capture selected components of CRS rather than its full multifactorial spectrum [46]. Third, while the sample size was adequate for the primary analyses, it limited statistical power for more granular subgroup assessments that could identify phenotypes with differential susceptibility to 5-OP accumulation. Finally, comprehensive tissue- and region-resolved assessment of glutathione flux and intracellular redox state remains technically challenging, limiting our mechanistic interpretation to OPLAH expression patterns rather than direct quantification of glutathione-cycle flux across all renal compartments. Circulating proteomics does not directly capture intracellular glutathione-cycle enzymes, necessitating renal enzyme-activity and tissue-level oxidative stress measurements as the subsequent natural steps to characterize the pathway.

## 5 Conclusions

This study provides evidence that circulating 5-OP predicts adverse outcomes in HF and is robustly associated with TGF-α, consistent with an interplay between renal 5-OP handling and growth-factor signaling that may be relevant to cardiorenal syndrome. In particular, our results support emerging evidence that oxidative stress markers such as 5-OP may serve as prognostic biomarkers and as mechanistically informative metabolites that motivate therapeutic hypothesis testing in both, cardiac and renal disease [16]. Looking ahead, further research should validate the prognostic value of 5-oxoproline in CRS through large-scale, multicenter studies with extended follow-up. Additionally, it is imperative to investigate the compensatory mechanisms that maintain glutamate homeostasis when OPLAH expression is altered and to explore therapeutic strategies—such as antioxidant supplementation or OPLAH enzyme replacement—that target the gamma-glutamyl cycle. In the future, integrating 5-oxoproline measurements with established biomarkers like NT-proBNP and kidney injury molecule-1 (KIM-1) may improve clinical decision-making and ultimately optimize patient care in cardiorenal syndrome.

## CRediT authorship contribution statement

**Antonio Esquivel-Gaytan:** Conceptualization, Data curation, Formal analysis, Investigation, Methodology, Project administration, Visualization, Writing – original draft, Writing – review & editing. **Ali A. Al-Mubarak:** Data curation, Formal analysis, Methodology, Writing – review & editing. **Oana Sorop:** Data curation, Methodology, Resources, Writing – review & editing. **Daphne Merkus:** Formal analysis, Methodology, Resources, Writing – review & editing. **Adriaan A. Voors:** Funding acquisition, Resources, Writing – review & editing. **Herman H.W. Silljé:** Resources, Supervision, Writing – review & editing. **Dirk J. Duncker:** Investigation, Methodology, Resources, Writing – review & editing. **Peter van der Meer:** Funding acquisition, Resources, Supervision, Writing – original draft, Writing – review & editing. **Nils Bomer:** Conceptualization, Investigation, Methodology, Supervision, Writing – original draft, Writing – review & editing.

## Funding

This study was supported by the Dutch Cardiovascular Alliance: an initiative with the support of the Dutch Heart Foundation (grant 2020B008 RECONNEXT).

Dutch Heart Foundation: ESCAPE-HF, 2018B012.

## Declaration of competing interests

The authors declare the following financial interests/personal relationships which may be considered as potential competing interests: Peter van der Meer reports financial support was provided by Netherlands Heart Foundation. Dirk J. Duncker reports financial support was provided by Netherlands Heart Foundation. The employer of AAV received consultancy fees and/or research support from Adrenomed, Anacardio, Armgo, AstraZeneca, Bayer AG, BMS, Boehringer Ingelheim, Cardurion, Corteria, EliLilly, Merck, Moderna, Novartis, Novo Nordisk, SalubrisBio. The employer of DJD received research support from Cardiac Booster, Idris Oncology, Medtronic, Oxitope Pharma. If there are other authors, they declare that they have no known competing financial interests or personal relationships that could have appeared to influence the work reported in this paper.

## References

[1] McDonagh T.A., Metra M., Adamo M., Gardner R.S., Baumbach A., Böhm M., Burri H., Butler J., Čelutkienė J., Chioncel O., Cleland J.G.F., Coats A.J.S., Crespo-Leiro M.G., Farmakis D., Gilard M., Heymans S., Hoes A.W., Jaarsma T., Jankowska E.A., Lainscak M., Lam C.S.P., Lyon A.R., V McMurray J.J., Mebazaa A., Mindham R., Muneretto C., Francesco Piepoli M., Price S., Rosano G.M.C., Ruschitzka F., Kathrine Skibelund A., de Boer R.A., Christian Schulze P., Abdelhamid M., Aboyans V., Adamopoulos S., Anker S.D., Arbelo E., Asteggiano R., Bauersachs J., Bayes-Genis A., Borger M.A., Budts W., Cikes M., Damman K., Delgado V., Dendale P., Dilaveris P., Drexel H., Ezekowitz J., Falk V., Fauchier L., Filippatos G., Fraser A., Frey N., Gale C.P., Gustafsson F., Harris J., Iung B., Janssens S., Jessup M., Konradi A., Kotecha D., Lambrinou E., Lancellotti P., Landmesser U., Leclercq C., Lewis B.S., Leyva F., Linhart A., Løchen M.-L., Lund L.H., Mancini D., Masip J., Milicic D., Mueller C., Nef H., Nielsen J.-C., Neubeck L., Noutsias M., Petersen S.E., Sonia Petronio A., Ponikowski P., Prescott E., Rakisheva A., Richter D.J., Schlyakhto E., Seferovic P., Senni M., Sitges M., Sousa-Uva M., Tocchetti C.G., Touyz R.M., Tschoepe C., Waltenberger J., Adamo M., Baumbach A., Böhm M., Burri H., Čelutkienė J., Chioncel O., Cleland J.G.F., Coats A.J.S., Crespo-Leiro M.G., Farmakis D., Gardner R.S., Gilard M., Heymans S., Hoes A.W., Jaarsma T., Jankowska E.A., Lainscak M., Lam C.S.P., Lyon A.R., V McMurray J.J., Mebazaa A., Mindham R., Muneretto C., Piepoli M.F., Price S., Rosano G.M.C., Ruschitzka F., Skibelund A.K. (2021). 2021 ESC guidelines for the diagnosis and treatment of acute and chronic heart failure. Eur. Heart J..

[2] Heidenreich P.A., Bozkurt B., Aguilar D., Allen L.A., Byun J.J., Colvin M.M., Deswal A., Drazner M.H., Dunlay S.M., Evers L.R., Fang J.C., Fedson S.E., Fonarow G.C., Hayek S.S., Hernandez A.F., Khazanie P., Kittleson M.M., Lee C.S., Link M.S., Milano C.A., Nnacheta L.C., Sandhu A.T., Stevenson L.W., Vardeny O., Vest A.R., Yancy C.W. (2022). AHA/ACC/HFSA guideline for the management of heart failure: a report of the American college of cardiology/American heart association joint committee on clinical practice guidelines. Circulation.

[3] Giam B., Kaye D.M., Rajapakse N.W. (2016). Role of renal oxidative stress in the pathogenesis of the cardiorenal syndrome. Heart Lung Circ..

[4] Takahama H., Kitakaze M. (2017). Pathophysiology of cardiorenal syndrome in patients with heart failure: potential therapeutic targets. Am. J. Physiol. Heart Circ. Physiol..

[5] Brouwers F.P., De Boer R.A., Van Der Harst P., Voors A.A., Gansevoort R.T., Bakker S.J., Hillege H.L., Van Veldhuisen D.J., Van Gilst W.H. (2013). Incidence and epidemiology of new onset heart failure with preserved vs. reduced ejection fraction in a community-based cohort: 11-year follow-up of PREVEND. Eur. Heart J..

[6] ter Maaten J.M., Damman K., Verhaar M.C., Paulus W.J., Duncker D.J., Cheng C., van Heerebeek L., Hillege H.L., Lam C.S.P., Navis G., Voors A.A. (2016). Connecting heart failure with preserved ejection fraction and renal dysfunction: the role of endothelial dysfunction and inflammation. Eur. J. Heart Fail..

[7] Rubattu S., Mennuni S., Testa M., Mennuni M., Pierelli G., Pagliaro B., Gabriele E., Coluccia R., Autore C., Volpe M. (2013). Pathogenesis of chronic cardiorenal syndrome: is there a role for oxidative stress?. Int. J. Mol. Sci..

[8] Shah B.N., Greaves K., Rosner M.H. (2010). The cardiorenal syndrome: a review. Int. J. Nephrol..

[9] Tsutsui H., Kinugawa S., Matsushima S. (2011). Oxidative stress and heart failure. Am. J. Physiol. Heart Circ. Physiol..

[10] Duni A., Liakopoulos V., Roumeliotis S., Peschos D., Dounousi E. (2019). Oxidative stress in the pathogenesis and evolution of chronic kidney disease: untangling ariadne's thread. Int. J. Mol. Sci..

[11] Irazabal M.V., Torres V.E. (2020). Reactive oxygen species and redox signaling in chronic kidney disease. Cells.

[12] Mitrić A., Castellano I. (2023). Targeting gamma-glutamyl transpeptidase: a pleiotropic enzyme involved in glutathione metabolism and in the control of redox homeostasis. Free Radic. Biol. Med..

[13] Al-Mubarak A.A., Esquivel-Gaytan A., Silljé H.H.W., van der Meer P., Bomer N. (2025). Glutathione deficiency and heart failure: a systematic review of human and animal evidence. Adv. Redox Res..

[14] Van der Werf P., Orlowski M., Meister A. (1971). Enzymatic conversion of 5-Oxo-L-Proline (L-Pyrrolidone carboxylate) to L-Glutamate coupled with cleavage of adenosine triphosphate to adenosine diphosphate, a reaction in the γ-Glutamyl cycle. Proc. Natl. Acad. Sci. USA.

[15] Orlowski M., Meister A. (1970). The γ-Glutamyl cycle: a possible transport system for amino acids. Proc. Natl. Acad. Sci..

[16] van der Pol A., Gil A., Tromp J., Silljé H.H.W., van Veldhuisen D.J., Voors A.A., Hoendermis E.S., Grote Beverborg N., Schouten E.-M., de Boer R.A., Bischoff R., van der Meer P. (2018). OPLAH ablation leads to accumulation of 5-oxoproline, oxidative stress, fibrosis, and elevated fillings pressures: a murine model for heart failure with a preserved ejection fraction. Cardiovasc. Res..

[17] Van Der Pol A., Gil A., Silljé H.H.W., Tromp J., Ovchinnikova E.S., Vreeswijk-Baudoin I., Hoes M., Domian I.J., Van De Sluis B., Van Deursen J.M., Voors A.A., Van Veldhuisen D.J., Van Gilst W.H., Berezikov E., Van Der Harst P., De Boer R.A., Bischoff R., Van Der Meer P. (2017). Accumulation of 5-oxoproline in myocardial dysfunction and the protective effects of OPLAH. Sci. Transl. Med..

[18] Griffith O.W., Meister A. (1979). Glutathione: interorgan translocation, turnover, and metabolism. Proc. Natl. Acad. Sci. USA.

[19] Sekura R., Meister A. (1974). Glutathione turnover in the kidney; considerations relating to the γ-Glutamyl cycle and the transport of amino acids. Proc. Natl. Acad. Sci. USA.

[20] Yu B., Zheng Y., Nettleton J.A., Alexander D., Coresh J., Boerwinkle E. (2014). Serum metabolomic profiling and incident ckd among African Americans. Clin. J. Am. Soc. Nephrol..

[21] Kimura T., Yasuda K., Yamamoto R., Soga T., Rakugi H., Hayashi T., Isaka Y. (2016). Identification of biomarkers for development of end-stage kidney disease in chronic kidney disease by metabolomic profiling. Sci. Rep..

[22] Voors A.A., Ouwerkerk W., Zannad F., van Veldhuisen D.J., Samani N.J., Ponikowski P., Ng L.L., Metra M., ter Maaten J.M., Lang C.C., Hillege H.L., van der Harst P., Filippatos G., Dickstein K., Cleland J.G., Anker S.D., Zwinderman A.H. (2017). Development and validation of multivariable models to predict mortality and hospitalization in patients with heart failure. Eur. J. Heart Fail..

[23] Voors A.A., Anker S.D., Cleland J.G., Dickstein K., Filippatos G., van der Harst P., Hillege H.L., Lang C.C., ter Maaten J.M., Ng L., Ponikowski P., Samani N.J., van Veldhuisen D.J., Zannad F., Zwinderman A.H., Metra M. (2016). A systems BIOlogy study to TAilored treatment in chronic heart failure: rationale, design, and baseline characteristics of BIOSTAT-CHF. Eur. J. Heart Fail..

[24] Breheny P., Huang J. (2011). Coordinate descent algorithms for nonconvex penalized regression, with applications to biological feature selection. Ann. Appl. Stat..

[25] Abram S.V., Helwig N.E., Moodie C.A., DeYoung C.G., MacDonald A.W., Waller N.G. (2016). Bootstrap enhanced penalized regression for variable selection with neuroimaging data. Front. Neurosci..

[26] Jiang H., Zheng W., Dong Y. (2021). Sparse and robust estimation with ridge minimax concave penalty. Inf. Sci..

[27] Lu M., Zhou J., Naylor C., Kirkpatrick B.D., Haque R., Petri W.A., Ma J.Z. (2017). Application of penalized linear regression methods to the selection of environmental enteropathy biomarkers. Biomark. Res..

[28] Zhang C.H. (2010). Nearly unbiased variable selection under minimax concave penalty.

[29] Benjamini Y., Hochberg Y. (1995). Controlling the false discovery rate: a practical and powerful approach to multiple testing. J. Roy. Stat. Soc. B.

[30] Sorop O., Heinonen I., Van Kranenburg M., Van De Wouw J., De Beer V.J., Nguyen I.T.N., Octavia Y., Van Duin R.W.B., Stam K., Van Geuns R.J., Wielopolski P.A., Krestin G.P., Van Den Meiracker A.H., Verjans R., Van Bilsen M., Danser A.H.J., Paulus W.J., Cheng C., Linke W.A., Joles J.A., Verhaar M.C., Van Der Velden J., Merkus D., Duncker D.J. (2018). Multiple common comorbidities produce left ventricular diastolic dysfunction associated with coronary microvascular dysfunction, oxidative stress, and myocardial stiffening. Cardiovasc. Res..

[31] van de Wouw J., Sorop O., van Drie R.W.A., van Duin R.W.B., Nguyen I.T.N., Joles J.A., Verhaar M.C., Merkus D., Duncker D.J. (2020). Perturbations in myocardial perfusion and oxygen balance in swine with multiple risk factors: a novel model of ischemia and no obstructive coronary artery disease. Basic Res. Cardiol..

[32] Guo X., Jiang M., Tao Z., Dai H., Wu C., Wang Y., Wang Z., Wang X., Zhang Z., Qian K., Zeng S., Bei Y., Pu J. (2025). 5-Oxoproline prevents doxorubicin-induced cardiotoxicity and tumor growth. Redox Biol..

[33] Heuer J.G., Harlan S.M., Yang D.D., Jaqua D.L., Boyles J.S., Wilson J.M., Heinz-Taheny K.M., Sullivan J.M., Wei T., Qian H.R., Witcher D.R., Breyer M.D. (2017). Role of TGF-alpha in the progression of diabetic kidney disease. Am. J. Physiol. Renal Physiol..

[34] Tang J., Liu N., Zhuang S. (2013). Role of epidermal growth factor receptor in acute and chronic kidney injury. Kidney Int..

[35] Forrester S.J., Kawai T., O'Brien S., Thomas W., Harris R.C., Eguchi S. (2016). Epidermal growth factor receptor transactivation: mechanisms, pathophysiology, and potential therapies in the cardiovascular system. Annu. Rev. Pharmacol. Toxicol..

[36] Bienaimé F., Muorah M., Metzger M., Broeuilh M., Houiller P., Flamant M., Haymann J.P., Vonderscher J., Mizrahi J., Friedlander G., Stengel B., Terzi F., Vrtovsnik F., Daugas E., Vidal-Petiot E., Jacquot C., Karras A., Roueff S., Thervet E., Houillier P., Courbebaisse M., Eladari D., Maruani Gérard, Urena-Torres P., Boffa J.J., Ronco P., Fessi H., Rondeau E., Letavernier E., Tabibzadeh N. (2023). Combining robust urine biomarkers to assess chronic kidney disease progression. EBioMedicine.

[37] Laouari D., Burtin M., Phelep A., Martino C., Pillebout E., Montagutelli X., Friedlander G., Terzi F. (2011). TGF-α mediates genetic susceptibility to chronic kidney disease. J. Am. Soc. Nephrol..

[38] Bettelli E., Carrier Y., Gao W., Korn T., Strom T.B., Oukka M., Weiner H.L., Kuchroo V.K. (2006). Reciprocal developmental pathways for the generation of pathogenic effector TH17 and regulatory T cells. Nature.

[39] Van Linthout S., Tschöpe C. (2017). Inflammation - Cause or consequence of heart failure or both?. Curr. Heart Fail. Rep..

[40] Cai Y.H., Ma Z.J., Lu X.Y., Le He E., You M.Y. (2016). Study on the effect and mechanism of the dysfunction of CD4+ T cells in the disease process of chronic cardiac failure. Asian Pac. J. Trop. Med..

[41] Li N., Bian H., Zhang J., Li X., Ji X., Zhang Y. (2010). The Th17/Treg imbalance exists in patients with heart failure with normal ejection fraction and heart failure with reduced ejection fraction. Clin. Chim. Acta.

[42] Ronco C., Bellomo R., Kellum J.A. (2019). Acute kidney injury. Lancet.

[43] Lash L.H. (2005). Role of glutathione transport processes in kidney function. Toxicol. Appl. Pharmacol..

[44] Luyckx V.A., Brenner B.M. (2010). The clinical importance of nephron mass. J. Am. Soc. Nephrol..

[45] Brisco M.A., Testani J.M. (2014). Novel renal biomarkers to assess cardiorenal syndrome, curr. Heart fail. Rep.

[46] Zhou B., Zhao J., Li D. (2024). A new animal model of cardiorenal syndrome could be established by inducing heart failure through coronary artery ligation in spontaneously hypertensive rats. Sci. Rep..

