# Supplementary material for: Elevated 5-oxoproline levels and adverse outcomes in heart failure: Association with renal cortical OPLAH loss in a multi-comorbidity model
Source: Redox Biol. 2026 Aug 17;96:104354. doi: 10.1016/j.redox.2026.104354 (PMC13520085; doi:10.1016/j.redox.2026.104354)
Supplement: Supplementary file 1 [file mmc1.docx]

# Supplementary Data

**Figure S1: Permutation Test**

Histogram showing the null distribution of TGF-α selection probabilities generated from N = 1000 outcome permutations (5-oxoproline), with stability selection rerun for each permutation using identical parameters (B = 100, q = 15). The observed TGF-α selection probability (π = 0.70) is overlaid. The dashed line denotes the 95th percentile of the null distribution. With 0/1000 exceedances, the empirical permutation p-value is ≤ 0.001 (Monte-Carlo resolution 0.001; p = 0.001 using the +1 correction).

**Figure S2. Comparison of permutation test null distributions for TGF-α selection probability**

Two complementary permutation approaches were used to assess the statistical significance of TGF-α selection. The TGF-α–specific test compares the observed TGF-α selection probability against its own null distribution generated by randomly permuting the outcome and re-running stability selection with identical parameters (N = 1000 permutations; B = 100; q = 15). With N = 1000 permutations, the Monte-Carlo resolution is 0.001; therefore, 0/1000 exceedances corresponds to an empirical p-value ≤ 0.001 (or p = 0.001 using the +1 correction). The global test compares the observed TGF-α probability against the distribution of the maximum selection probability under the null (familywise-style control), yielding p = 0.183, consistent with a conservative comparison.

**Figure S3. Targeted post hoc re-analysis of circulating tissue-remodeling/renal-injury and redox/stress-related proteins versus 5-oxoproline (n = 823).** Pearson correlations (points) with Fisher 95% confidence intervals (bars) for each protein against circulating 5-OP. Colours denote pre-defined groups: lead marker TGF-α (black), tissue-remodeling/renal-injury-related proteins (red), redox/stress-related proteins (blue), and canonical panel matrix/fibrosis markers shown for context but not part of either group (grey). Filled points with a black outline are significant after Benjamini-Hochberg correction across all 355 panel proteins (BH-adjusted P < 0.05). OLR1/LOX-1 denotes the oxidized-LDL receptor-1 (not lysyl oxidase).

**Table S1. List of Abbreviations Used in Main Text**

| Abbreviation | Full Term |
| --- | --- |
| ACE | Angiotensin Converting Enzyme |
| ARB | Angiotensin Receptor Blocker |
| BB | Beta Blocker |
| BNP | brain natriuretic peptide |
| CKD | chronic kidney disease |
| CRS | cardiorenal syndrome |
| DM | diabetes mellitus |
| eGFR | estimated glomerular filtration rate |
| EGFR | epidermal growth factor receptor |
| ESI | electrospray ionization |
| GSH | Glutathione |
| GGC | gamma-glutamyl cycle |
| HF | heart failure |
| HFD | high-fat diet |
| HFpEF | heart failure with preserved ejection fraction |
| HT | Hypertension |
| LC-MS | liquid chromatography-mass spectrometry |
| LV | left ventricle |
| LVEF | left ventricular ejection fraction |
| MCP | minimax concave penalty |
| MRM | multiple reaction monitoring |
| NT-proBNP | n-terminal pro-brain natriuretic peptide |
| OPLAH | 5-oxoprolinase |
| 5-OP | 5-oxoproline |
| PEA | proximity extension assay |
| ROS | reactive oxygen species |
| TGF-α | transforming growth factor alpha |

**Table S2. Association of circulating 5‑oxoproline with 2‑year composite outcome (HF hospitalization or death) in BIOSTAT‑CHF, stratified by CKD**

| Contrast | n (events) | HR (95 % CI) | P |
| --- | --- | --- | --- |
| Non‑CKD (eGFR ≥ 60) | 381 (90) | 1.17 (0.96–1.42) | 0.12 |
| CKD (eGFR < 60) | 333 (127) | 1.25 (1.05–1.48) | 0.012 |
| Main effect: CKD vs non‑CKD† | 714 (217) | 1.62 (1.19–2.13) | 0.002 |
| Interaction: CKD × 5‑OP slope‡ | — | 1.07 | 0.63 |

Model covariates: age, sex, body‑mass index, left‑ventricular ejection fraction, and NT‑proBNP (central lab).
Endpoint: first heart‑failure hospitalization or all‑cause death, censored at 2 years.

† *CKD main effect evaluated at log5op_z = 0 (cohort mean).*
‡ *Interaction (Stata c.log5op_z#1.CKD); CKD-specific slopes derived as exp(β) and exp(β+β_int).*

**Table S3. Adjusted selection probabilities after residualization by clinical covariates**

| **Biomarker** | **Adjusted_Probability** |
| --- | --- |
| **P01135_TGF_alpha** | 0.6 |
| **Q9Y3P8_SIT1** | 0.5 |
| **P09237_MMP7** | 0.49 |
| **Q8IW75_SERPINA12** | 0.46 |
| **Q04759_PRKCQ** | 0.42 |
| **npx_177_t_pa** | 0.42 |
| **P05231_IL6** | 0.39 |
| **Q9UJ71_CD207** | 0.38 |
| **O43736_ITM2A** | 0.31 |
| **P19883_FS** | 0.31 |
| **P21980_TGM2** | 0.31 |
| **Q9UKR3_KLK13** | 0.3 |
| **Q04760_GLO1** | 0.24 |
| **P35218_CA5A** | 0.23 |
| **P35052_GPC1** | 0.22 |
| **P23229_ITGA6** | 0.22 |
| **npx_113_selp** | 0.21 |
| **Q9UKX5_ITGA11** | 0.2 |
| **Q13421_MSLN** | 0.19 |
| **P58499_FAM3B** | 0.19 |
| **npx_107_mmp_9** | 0.19 |
| **P35968_VEGFR_2** | 0.17 |
| **P07949_RET** | 0.17 |
| **Q99895_CTRC** | 0.17 |
| **Q9UHC6_CNTNAP2** | 0.16 |
| **Q14512_FGF_BP1** | 0.15 |
| **Q03431_PTH1R** | 0.15 |
| **P05113_IL5** | 0.15 |
| **Q9HBG7_LY9** | 0.14 |
| **Q9P0G3_hK14** | 0.14 |
| **O00622_CYR61** | 0.13 |
| **Q8WXI8_CLEC4D** | 0.13 |
| **npx_152_ep_cam** | 0.13 |
| **npx_186_mmp_3** | 0.13 |
| **P18510_IL_1ra** | 0.12 |
| **P35475_IDUA** | 0.11 |
| **P41439_FR_gamma** | 0.1 |
| **P27352_GIF** | 0.1 |
| **Q9BYF1_ACE2** | 0.1 |
| **O14836_TNFRSF13B** | 0.1 |
| **npx_172_casp_3** | 0.1 |
| **P21589_5__NT** | 0.09 |
| **Q9UBG3_CRNN** | 0.09 |
| **Q9NP99_TREM1** | 0.09 |
| **P35318_ADM** | 0.09 |
| **P01833_PIgR** | 0.09 |
| **npx_169_il_1rt2** | 0.09 |
| **Q29983_Q29980_MIC_A_B** | 0.08 |
| **Q99717_MAD_homolog_5** | 0.08 |
| **P63241_EIF5A** | 0.08 |
| **Q9Y2J8_PADI2** | 0.08 |
| **P25116_PAR_1** | 0.08 |
| **npx_149_ccl22** | 0.08 |
| **Q8TE58_ADAM_TS_15** | 0.07 |
| **Q13241_KLRD1** | 0.07 |
| **P27540_ARNT** | 0.07 |
| **P35442_THBS2** | 0.07 |
| **Q8IYS5_hOSCAR** | 0.07 |
| **npx_144_igfbp_1** | 0.07 |
| **npx_168_gal_4** | 0.07 |
| **P78325_ADAM_8** | 0.06 |
| **P32970_CD70** | 0.06 |
| **P06731_CEACAM5** | 0.06 |
| **P14317_HCLS1** | 0.06 |
| **Q6UXB4_CLEC4G** | 0.06 |
| **P05412_JUN** | 0.06 |
| **P48740_MASP1** | 0.06 |
| **Q76LX8_ADAM_TS13** | 0.06 |
| **Q14116_IL18** | 0.06 |
| **O43915_VEGFD** | 0.06 |
| **npx_102_ldl_receptor** | 0.06 |
| **npx_132_ccl24** | 0.06 |
| **npx_138_dlk_1** | 0.06 |
| **npx_166_cpa1** | 0.06 |
| **npx_167_jam_a** | 0.06 |
| **npx_176_st2** | 0.06 |
| **O75475_PSIP1** | 0.05 |
| **P34130_NTF4** | 0.05 |
| **Q6ZUJ8_PIK3AP1** | 0.05 |
| **P22004_BMP_6** | 0.05 |
| **P09341_CXCL1** | 0.05 |
| **P00797_REN** | 0.05 |
| **Q96D42_KIM1** | 0.05 |
| **P10147_CCL3** | 0.05 |
| **Q9UEW3_MARCO** | 0.05 |
| **P16860_BNP** | 0.05 |
| **npx_192_igfbp_2** | 0.05 |
| **P26447_S100A4** | 0.04 |
| **P01298_PPY** | 0.04 |
| **Q9BXY4_RSPO3** | 0.04 |
| **Q15661_TPSAB1** | 0.04 |
| **Q9UNE0_EDAR** | 0.04 |
| **Q8N608_DPP10** | 0.04 |
| **Q14435_GALNT3** | 0.04 |
| **P10747_CD28** | 0.04 |
| **P49763_PGF** | 0.04 |
| **Q8NEV9_Q14213_IL_27** | 0.04 |
| **P51161_GT** | 0.04 |
| **P09874_PARP_1** | 0.04 |
| **Q9UJM8_HAOX1** | 0.04 |
| **npx_187_rarres2** | 0.04 |
| **P16870_CPE** | 0.03 |
| **P20718_GZMH** | 0.03 |
| **P09382_Gal_1** | 0.03 |
| **Q9NQ30_ESM_1** | 0.03 |
| **P30048_PRDX3** | 0.03 |
| **Q9C035_TRIM5** | 0.03 |
| **O60449_LY75** | 0.03 |
| **P28845_HSD11B1** | 0.03 |
| **P16455_MGMT** | 0.03 |
| **P52823_STC1** | 0.03 |
| **Q9Y6Q6_TNFRSF11A** | 0.03 |
| **Q9BQR3_PRSS27** | 0.03 |
| **P01241_GH** | 0.03 |
| **Q13219_PAPPA** | 0.03 |
| **Q16698_DECR1** | 0.03 |
| **P09601_HO_1** | 0.03 |
| **P26022_PTX3** | 0.03 |
| **O94907_Dkk_1** | 0.03 |
| **P12104_FABP2** | 0.03 |
| **npx_129_fabp4** | 0.03 |
| **npx_133_tr** | 0.03 |
| **npx_148_tr_ap** | 0.03 |
| **npx_156_mmp_2** | 0.03 |
| **npx_183_col1a1** | 0.03 |
| **O95274_LYPD3** | 0.02 |
| **O00592_PODXL** | 0.02 |
| **P21583_SCF** | 0.02 |
| **P09958_FURIN** | 0.02 |
| **Q14956_GPNMB** | 0.02 |
| **Q16790_CAIX** | 0.02 |
| **P14210_HGF** | 0.02 |
| **O15455_TLR3** | 0.02 |
| **O75144_ICOSLG** | 0.02 |
| **Q8WXI7_MUC_16** | 0.02 |
| **Q6BAA4_FCRLB** | 0.02 |
| **P04083_ANXA1** | 0.02 |
| **Q8WTT0_CLEC4C** | 0.02 |
| **Q8NHJ6_LILRB4** | 0.02 |
| **P08727_KRT19** | 0.02 |
| **Q6EIG7_CLEC6A** | 0.02 |
| **Q9BWV1_BOC** | 0.02 |
| **P47992_XCL1** | 0.02 |
| **P31997_CEACAM8** | 0.02 |
| **Q14242_PSGL_1** | 0.02 |
| **Q92583_CCL17** | 0.02 |
| **P06858_LPL** | 0.02 |
| **O00253_AGRP** | 0.02 |
| **Q9BQ51_PD_L2** | 0.02 |
| **P04792_HSP_27** | 0.02 |
| **npx_116_cd163** | 0.02 |
| **npx_120_mepe** | 0.02 |
| **npx_142_il_6ra** | 0.02 |
| **npx_174_cpb1** | 0.02 |
| **npx_175_chi3l1** | 0.02 |
| **npx_178_scgb3a2** | 0.02 |
| **O75888_TNFSF13** | 0.01 |
| **O95407_TNFRSF6B** | 0.01 |
| **P09326_CD48** | 0.01 |
| **P06756_ITGAV** | 0.01 |
| **Q9UBX7_hK11** | 0.01 |
| **P48307_TFPI_2** | 0.01 |
| **O60259_hK8** | 0.01 |
| **P21860_ERBB3** | 0.01 |
| **P18084_ITGB5** | 0.01 |
| **O60911_CTSV** | 0.01 |
| **O43895_XPNPEP2** | 0.01 |
| **Q15303_ERBB4** | 0.01 |
| **P21741_MK** | 0.01 |
| **Q9NS68_TNFRSF19** | 0.01 |
| **P56279_TCL1A** | 0.01 |
| **P43489_TNFRSF4** | 0.01 |
| **O95388_WISP_1** | 0.01 |
| **O43927_CXCL13** | 0.01 |
| **P16278_GLB1** | 0.01 |
| **Q13574_DGKZ** | 0.01 |
| **Q9UMR7_CLEC4A** | 0.01 |
| **Q96DB9_FXYD5** | 0.01 |
| **P50135_HNMT** | 0.01 |
| **P51671_CCL11** | 0.01 |
| **Q96P31_FCRL3** | 0.01 |
| **P22301_IL10** | 0.01 |
| **O14867_BACH1** | 0.01 |
| **Q96PD2_DCBLD2** | 0.01 |
| **P48061_CXCL12** | 0.01 |
| **Q9UQV4_LAMP3** | 0.01 |
| **Q92844_TANK** | 0.01 |
| **P52294_KPNA1** | 0.01 |
| **P18627_LAG3** | 0.01 |
| **Q01151_CD83** | 0.01 |
| **Q13043_STK4** | 0.01 |
| **P13726_TF** | 0.01 |
| **O00182_Gal_9** | 0.01 |
| **Q9NSA1_FGF_21** | 0.01 |
| **P04179_SOD2** | 0.01 |
| **Q96IQ7_VSIG2** | 0.01 |
| **P02760_AMBP** | 0.01 |
| **Q14005_IL16** | 0.01 |
| **Q99523_SORT1** | 0.01 |
| **P31994_IgG_Fc_receptor_II_b** | 0.01 |
| **P07585_DCN** | 0.01 |
| **Q16651_PRSS8** | 0.01 |
| **Q9UK05_GDF_2** | 0.01 |
| **P40225_THPO** | 0.01 |
| **P07711_CTSL1** | 0.01 |
| **npx_103_itgb2** | 0.01 |
| **npx_108_ephb4** | 0.01 |
| **npx_110_opg** | 0.01 |
| **npx_112_tff3** | 0.01 |
| **npx_115_mcp_1** | 0.01 |
| **npx_127_cdh5** | 0.01 |
| **npx_128_tlt_2** | 0.01 |
| **npx_130_tfpi** | 0.01 |
| **npx_131_pai** | 0.01 |
| **npx_145_chit1** | 0.01 |
| **npx_161_pcsk9** | 0.01 |
| **npx_162_u_par** | 0.01 |
| **npx_179_egfr** | 0.01 |
| **npx_181_cd93** | 0.01 |
| **npx_190_pdgf_subunit_a** | 0.01 |
| **npx_194_pecam_1** | 0.01 |
| **P40222_TXLNA** | 0 |
| **P15692_VEGFA** | 0 |
| **P13688_CEACAM1** | 0 |
| **P01133_EGF** | 0 |
| **P18827_SYND1** | 0 |
| **P37173_TGFR_2** | 0 |
| **O14828_SCAMP3** | 0 |
| **P15260_IFN_gamma_R1** | 0 |
| **P50591_TRAIL** | 0 |
| **P08069_IGF1R** | 0 |
| **P04626_ERBB2** | 0 |
| **P09486_SPARC** | 0 |
| **Q13158_FADD** | 0 |
| **P50579_MetAP_2** | 0 |
| **Q96NY8_PVRL4** | 0 |
| **P48023_FASLG** | 0 |
| **P29317_EPHA2** | 0 |
| **Q9BYH1_SEZ6L** | 0 |
| **Q16674_MIA** | 0 |
| **P26842_CD27** | 0 |
| **P38936_CDKN1A** | 0 |
| **O00548_DLL1** | 0 |
| **P00519_ABL1** | 0 |
| **P07948_LYN** | 0 |
| **P08670_VIM** | 0 |
| **O95971_CD160** | 0 |
| **Q6UXB2_CXL17** | 0 |
| **P31949_S100A11** | 0 |
| **P15514_AREG** | 0 |
| **Q14508_WFDC2** | 0 |
| **P35916_VEGFR_3** | 0 |
| **Q9Y5W5_WIF_1** | 0 |
| **P10144_GZMB** | 0 |
| **P15328_FR_alpha** | 0 |
| **Q96SB3_PPP1R9B** | 0 |
| **Q05516_ZBTB16** | 0 |
| **Q9NWZ3_IRAK4** | 0 |
| **Q00978_IRF9** | 0 |
| **P51617_IRAK1** | 0 |
| **Q06830_PRDX1** | 0 |
| **P09038_FGF2** | 0 |
| **P30044_PRDX5** | 0 |
| **Q14203_DCTN1** | 0 |
| **Q15517_CDSN** | 0 |
| **Q12933_TRAF2** | 0 |
| **P19474_TRIM21** | 0 |
| **Q7Z6M3_MILR1** | 0 |
| **Q9GZT9_EGLN1** | 0 |
| **Q12968_NFATC3** | 0 |
| **Q04637_EIF4G1** | 0 |
| **Q13490_BIRC2** | 0 |
| **P35240_NF2** | 0 |
| **Q9HCM2_PLXNA4** | 0 |
| **Q9UQQ2_SH2B3** | 0 |
| **Q07065_CKAP4** | 0 |
| **O94992_HEXIM1** | 0 |
| **P78310_CXADR** | 0 |
| **P78362_SRPK2** | 0 |
| **O43597_SPRY2** | 0 |
| **O60880_SH2D1A** | 0 |
| **Q05084_ICA1** | 0 |
| **O00273_DFFA** | 0 |
| **Q6DN72_FCRL6** | 0 |
| **O76036_NCR1** | 0 |
| **Q8IU57_IFNLR1** | 0 |
| **Q9UN19_DAPP1** | 0 |
| **Q9BXN2_CLEC7A** | 0 |
| **O95786_DDX58** | 0 |
| **P42701_IL12RB1** | 0 |
| **P18564_ITGB6** | 0 |
| **P78410_BTN3A2** | 0 |
| **Q15389_ANGPT1** | 0 |
| **P29965_CD40_L** | 0 |
| **Q9NQ25_SLAMF7** | 0 |
| **P24394_IL_4RA** | 0 |
| **P12931_SRC** | 0 |
| **O00220_TNFRSF10A** | 0 |
| **O14763_TRAIL_R2** | 0 |
| **Q02763_TIE2** | 0 |
| **Q9HB29_IL1RL2** | 0 |
| **P01127_PDGF_subunit_B** | 0 |
| **Q8TAD2_IL_17D** | 0 |
| **P78380_LOX_1** | 0 |
| **Q15109_RAGE** | 0 |
| **Q9GZV9_FGF_23** | 0 |
| **Q9BUD6_SPON2** | 0 |
| **Q9UIB8_CD84** | 0 |
| **Q12866_MERTK** | 0 |
| **P07204_TM** | 0 |
| **P51888_PRELP** | 0 |
| **Q9UKP3_ITGB1BP2** | 0 |
| **Q99075_HB_EGF** | 0 |
| **P39900_MMP12** | 0 |
| **P41159_LEP** | 0 |
| **P01730_CD4** | 0 |
| **Q9Y6K9_NEMO** | 0 |
| **npx_101_tnfrsf14** | 0 |
| **npx_105_il_17ra** | 0 |
| **npx_106_tnf_r2** | 0 |
| **npx_109_il2_ra** | 0 |
| **npx_111_alcam** | 0 |
| **npx_117_gal_3** | 0 |
| **npx_118_grn** | 0 |
| **npx_122_plc** | 0 |
| **npx_123_ltbr** | 0 |
| **npx_124_notch_3** | 0 |
| **npx_125_timp4** | 0 |
| **npx_126_cntn1** | 0 |
| **npx_134_tnfrsf10c** | 0 |
| **npx_135_gdf_15** | 0 |
| **npx_136_sele** | 0 |
| **npx_139_spon1** | 0 |
| **npx_140_mpo** | 0 |
| **npx_141_cxcl16** | 0 |
| **npx_143_retn** | 0 |
| **npx_153_ap_n** | 0 |
| **npx_154_axl** | 0 |
| **npx_155_il_1rt1** | 0 |
| **npx_157_fas** | 0 |
| **npx_158_mb** | 0 |
| **npx_159_tnfsf13b** | 0 |
| **npx_163_opn** | 0 |
| **npx_164_ctsd** | 0 |
| **npx_165_pglyrp1** | 0 |
| **npx_170_shps_1** | 0 |
| **npx_171_ccl15** | 0 |
| **npx_173_upa** | 0 |
| **npx_180_igfbp_7** | 0 |
| **npx_182_il_18bp** | 0 |
| **npx_184_pon3** | 0 |
| **npx_185_ctsz** | 0 |
| **npx_188_icam_2** | 0 |
| **npx_193_vwf** | 0 |
| **npx_196_ccl16** | 0 |

**Table S4. Biomarker Associations with Circulating 5-Oxoproline Levels**

| Biomarker | Full name | Standardized coefficient* | Selection probability (π) |
| --- | --- | --- | --- |
| TGF-α | Transforming growth factor-α | 0.211 | 0.70 |
| MMP7 | Matrix metalloproteinase-7 | -0.040 | 0.55 |
| IL-6 | Interleukin-6 | -0.083 | 0.47 |
| SERPINA12 | Serpin A12 (Vaspin) | -0.047 | 0.46 |
| t-PA | Tissue plasminogen activator | 0.081 | 0.44 |
| RET | RET proto-oncogene | -0.014 | 0.36 |
| SIT1 | Signaling threshold regulating protein | 0.051 | 0.36 |
| FAM3B | FAM3 metabolism regulating signaling molecule B | -0.060 | 0.27 |
| CD207 | Langerin | -0.033 | 0.26 |
| FST | Follistatin | -0.032 | 0.26 |
| LY9 | Lymphocyte antigen 9 | -0.042 | 0.25 |
| PRKCQ | Protein kinase C theta | -0.026 | 0.25 |
| KLK13 | Kallikrein-13 | -0.027 | 0.24 |
| CA5A | Carbonic anhydrase 5A | -0.062 | 0.23 |
| GLO1 | Glyoxalase I | 0.118 | 0.22 |
| SELP | P-selectin | -0.094 | 0.22 |
| VEGFR-2 (KDR) | Vascular endothelial growth factor receptor 2 | -0.061 | 0.21 |
| TREM1 | Triggering receptor expressed on myeloid cells 1 | 0.126 | 0.21 |
| IDUA | Alpha-L-iduronidase | -0.017 | 0.21 |
| CRNN | Cornulin | -0.015 | 0.20 |

*Standardized coefficients from MCP-penalized regression. Positive coefficients indicate biomarkers that increase with higher 5-oxoproline levels; negative coefficients indicate inverse associations. TGF-α was the only biomarker meeting the pre-specified robustness threshold (π ≥ 0.70).

**Table S5: Pathway enrichment analysis of the top 20 biomarkers associated with circulating 5-oxoproline.**

| **Database** | **Term** | **P-value** | **Adjusted_P** | **Genes** |
| --- | --- | --- | --- | --- |
| **GO Biological Process** | Positive Regulation Of Interleukin-17 Production (GO:0032740) | 1.71E-06 | 7.07E-04 | ['IL6', 'PRKCQ', 'LY9'] |
| **GO Biological Process** | Regulation Of Interleukin-17 Production (GO:0032660) | 5.02E-06 | 7.07E-04 | ['IL6', 'PRKCQ', 'LY9'] |
| **GO Biological Process** | Membrane Protein Proteolysis (GO:0033619) | 5.02E-06 | 7.07E-04 | ['RET', 'MMP7', 'PRKCQ'] |
| **GO Biological Process** | T-helper 17 Cell Lineage Commitment (GO:0072540) | 1.99E-05 | 2.1E-03 | ['IL6', 'LY9'] |
| **GO Biological Process** | T-helper 17 Cell Differentiation (GO:0072539) | 2.65E-05 | 2.24E-03 | ['IL6', 'LY9'] |
| **GO Biological Process** | T-helper Cell Lineage Commitment (GO:0002295) | 4.25E-05 | 3E-03 | ['IL6', 'LY9'] |
| **GO Biological Process** | Defense Response To Gram-negative Bacterium (GO:0050829) | 5.51E-05 | 3.33E-03 | ['SELP', 'IL6', 'TREM1'] |
| **GO Biological Process** | Regulation Of Phosphatidylinositol 3-Kinase Signaling (GO:0014066) | 9.5E-05 | 5.02E-03 | ['CRNN', 'SELP', 'KDR'] |
| **GO Biological Process** | Positive Regulation Of Leukocyte Adhesion To Vascular Endothelial Cell (GO:1904996) | 1.28E-04 | 6.02E-03 | ['SELP', 'IL6'] |
| **GO Biological Process** | Calcium-Mediated Signaling Using Intracellular Calcium Source (GO:0035584) | 1.79E-04 | 7.55E-03 | ['SELP', 'KDR'] |
| **GO Molecular Function** | Serine-Type Endopeptidase Activity (GO:0004252) | 2.51E-04 | 6.22E-03 | ['MMP7', 'KLK13', 'PLAT'] |
| **GO Molecular Function** | Serine-Type Peptidase Activity (GO:0008236) | 3.66E-04 | 6.22E-03 | ['MMP7', 'KLK13', 'PLAT'] |
| **GO Molecular Function** | Transmembrane Receptor Protein Kinase Activity (GO:0019199) | 6.2E-04 | 7.02E-03 | ['RET', 'KDR'] |
| **GO Molecular Function** | Transmembrane Receptor Protein Tyrosine Kinase Activity (GO:0004714) | 1.13E-03 | 9.61E-03 | ['RET', 'KDR'] |
| **GO Molecular Function** | Growth Factor Activity (GO:0008083) | 3.23E-03 | 1.6E-02 | ['IL6', 'TGFA'] |
| **GO Molecular Function** | Receptor Ligand Activity (GO:0048018) | 3.75E-03 | 1.6E-02 | ['IL6', 'FAM3B', 'TGFA'] |
| **GO Molecular Function** | Growth Factor Receptor Binding (GO:0070851) | 4.09E-03 | 1.6E-02 | ['IL6', 'TGFA'] |
| **GO Molecular Function** | Endopeptidase Activity (GO:0004175) | 4.3E-03 | 1.6E-02 | ['MMP7', 'KLK13', 'PLAT'] |
| **GO Molecular Function** | Protein Tyrosine Kinase Activity (GO:0004713) | 4.35E-03 | 1.6E-02 | ['RET', 'KDR'] |
| **GO Molecular Function** | Calcium Ion Binding (GO:0005509) | 4.7E-03 | 1.6E-02 | ['RET', 'CRNN', 'SELP'] |
| **KEGG** | Malaria | 1.13E-03 | 7.47E-02 | ['SELP', 'IL6'] |
| **KEGG** | Non-small cell lung cancer | 2.33E-03 | 7.47E-02 | ['RET', 'TGFA'] |
| **KEGG** | Prostate cancer | 4.18E-03 | 7.47E-02 | ['TGFA', 'PLAT'] |
| **KEGG** | PI3K-Akt signaling pathway | 5.01E-03 | 7.47E-02 | ['IL6', 'KDR', 'TGFA'] |
| **KEGG** | Th17 cell differentiation | 5.06E-03 | 7.47E-02 | ['IL6', 'PRKCQ'] |
| **KEGG** | Insulin resistance | 5.15E-03 | 7.47E-02 | ['IL6', 'PRKCQ'] |
| **KEGG** | Fluid shear stress and atherosclerosis | 8.39E-03 | 1.04E-01 | ['KDR', 'PLAT'] |
| **KEGG** | Pathways in cancer | 1.52E-02 | 1.29E-01 | ['RET', 'IL6', 'TGFA'] |
| **KEGG** | Transcriptional misregulation in cancer | 1.55E-02 | 1.29E-01 | ['IL6', 'PLAT'] |
| **KEGG** | Nitrogen metabolism | 1.69E-02 | 1.29E-01 | ['CA5A'] |
| **Reactome** | MAPK1/MAPK3 Signaling R-HSA-5684996 | 1.46E-04 | 1.64E-02 | ['RET', 'IL6', 'TGFA', 'PRKCQ'] |
| **Reactome** | MAPK Family Signaling Cascades R-HSA-5683057 | 2.48E-04 | 1.64E-02 | ['RET', 'IL6', 'TGFA', 'PRKCQ'] |
| **Reactome** | Signal Transduction R-HSA-162582 | 1.68E-03 | 6.32E-02 | ['RET', 'IL6', 'MMP7', 'FST', 'KDR', 'TGFA', 'PLAT', 'PRKCQ'] |
| **Reactome** | RAF/MAP Kinase Cascade R-HSA-5673001 | 2.36E-03 | 6.32E-02 | ['RET', 'TGFA', 'PRKCQ'] |
| **Reactome** | Extra-nuclear Estrogen Signaling R-HSA-9009391 | 2.39E-03 | 6.32E-02 | ['MMP7', 'TGFA'] |
| **Reactome** | Signaling By Membrane-Tethered Fusions Of PDGFRA Or PDGFRB R-HSA-9673768 | 4.99E-03 | 8.12E-02 | ['KDR'] |
| **Reactome** | Regulation Of IGF Transport And Uptake By IGFBPs R-HSA-381426 | 6.63E-03 | 8.12E-02 | ['IL6', 'KLK13'] |
| **Reactome** | VEGF Binds To VEGFR Leading To Receptor Dimerization R-HSA-195399 | 7.97E-03 | 8.12E-02 | ['KDR'] |
| **Reactome** | Inhibition Of Signaling By Overexpressed EGFR R-HSA-5638303 | 7.97E-03 | 8.12E-02 | ['TGFA'] |
| **Reactome** | MAPK1 (ERK2) Activation R-HSA-112411 | 8.97E-03 | 8.12E-02 | ['IL6'] |
| **WikiPathway 2023 Human** | EGFR Tyrosine Kinase Inhibitor Resistance WP4806 | 7.74E-05 | 5.71E-03 | ['IL6', 'KDR', 'TGFA'] |
| **WikiPathway 2023 Human** | Cells And Molecules Involved In Local Acute Inflammatory Response WP4493 | 1.28E-04 | 5.71E-03 | ['SELP', 'IL6'] |
| **WikiPathway 2023 Human** | Extracellular Vesicles In The Crosstalk Of Cardiac Cells WP4300 | 1.61E-04 | 5.71E-03 | ['IL6', 'KDR'] |
| **WikiPathway 2023 Human** | LTF Danger Signal Response Pathway WP4478 | 1.61E-04 | 5.71E-03 | ['IL6', 'TREM1'] |
| **WikiPathway 2023 Human** | Fibrin Complement Receptor 3 Signaling Pathway WP4136 | 7.99E-04 | 1.78E-02 | ['IL6', 'PLAT'] |
| **WikiPathway 2023 Human** | Genes Controlling Nephrogenesis WP4823 | 8.76E-04 | 1.78E-02 | ['RET', 'KDR'] |
| **WikiPathway 2023 Human** | Chronic Hyperglycemia Impairment Of Neuron Function WP5283 | 9.58E-04 | 1.78E-02 | ['MMP7', 'PRKCQ'] |
| **WikiPathway 2023 Human** | Pluripotent Stem Cell Differentiation Pathway WP2848 | 1.04E-03 | 1.78E-02 | ['IL6', 'FST'] |
| **WikiPathway 2023 Human** | Vitamin B12 Metabolism WP1533 | 1.13E-03 | 1.78E-02 | ['IL6', 'PLAT'] |
| **WikiPathway 2023 Human** | Network Map Of SARS CoV 2 Signaling Pathway WP5115 | 1.27E-03 | 1.80E-02 | ['SELP', 'IL6', 'PRKCQ'] |
| **GO Cellular Component** | Clathrin-Coated Endocytic Vesicle Membrane (GO:0030669) | 2.08E-03 | 3.39E-02 | ['CD207', 'TGFA'] |
| **GO Cellular Component** | Clathrin-Coated Endocytic Vesicle (GO:0045334) | 3.23E-03 | 3.39E-02 | ['CD207', 'TGFA'] |
| **GO Cellular Component** | Clathrin-Coated Vesicle Membrane (GO:0030665) | 3.61E-03 | 3.39E-02 | ['CD207', 'TGFA'] |
| **GO Cellular Component** | Serine Protease Inhibitor Complex (GO:0097180) | 4.99E-03 | 3.39E-02 | ['PLAT'] |
| **GO Cellular Component** | Platelet Dense Granule Membrane (GO:0031088) | 4.99E-03 | 3.39E-02 | ['SELP'] |
| **GO Cellular Component** | Endocytic Vesicle Membrane (GO:0030666) | 1.09E-02 | 6.16E-02 | ['CD207', 'TGFA'] |
| **GO Cellular Component** | Platelet Alpha Granule Membrane (GO:0031092) | 1.59E-02 | 7.72E-02 | ['SELP'] |
| **GO Cellular Component** | Platelet Dense Granule (GO:0042827) | 1.98E-02 | 8.42E-02 | ['SELP'] |
| **GO Cellular Component** | Aggresome (GO:0016235) | 3.44E-02 | 1.26E-01 | ['PRKCQ'] |
| **GO Cellular Component** | Early Endosome (GO:0005769) | 3.72E-02 | 1.26E-01 | ['CD207', 'KDR'] |
| **MSigDB Hallmark 2020** | Coagulation | 8.28E-03 | 4.20E-02 | ['MMP7', 'PLAT'] |
| **MSigDB Hallmark 2020** | UV Response Up | 1.07E-02 | 4.20E-02 | ['RET', 'IL6'] |
| **MSigDB Hallmark 2020** | Apoptosis | 1.11E-02 | 4.20E-02 | ['IL6', 'PLAT'] |
| **MSigDB Hallmark 2020** | Glycolysis | 1.68E-02 | 4.20E-02 | ['IDUA', 'TGFA'] |
| **MSigDB Hallmark 2020** | Allograft Rejection | 1.68E-02 | 4.20E-02 | ['IL6', 'SIT1'] |
| **MSigDB Hallmark 2020** | KRAS Signaling Dn | 1.68E-02 | 4.20E-02 | ['IDUA', 'CD207'] |
| **MSigDB Hallmark 2020** | Interferon Gamma Response | 1.68E-02 | 4.20E-02 | ['SELP', 'IL6'] |
| **MSigDB Hallmark 2020** | Complement | 1.68E-02 | 4.20E-02 | ['IL6', 'PLAT'] |
| **MSigDB Hallmark 2020** | IL-6/JAK/STAT3 Signaling | 8.35E-02 | 1.82E-01 | ['IL6'] |
| **MSigDB Hallmark 2020** | IL-2/STAT5 Signaling | 1.81E-01 | 1.82E-01 | ['SELP'] |
| **STRING DISEASES** | Thrombosis | 1.78E-06 | 8.20E-03 | ['PLAT', 'SELP', 'IL6'] |
| **STRING DISEASES** | Carotid artery disease | 2.73E-05 | 3.36E-02 | ['PLAT', 'SELP'] |
| **STRING DISEASES** | Cerebral infarction | 1.46E-05 | 3.36E-02 | ['PLAT', 'IL6'] |
| **STRING DISEASES** | Cerebrovascular disease | 1.60E-05 | 3.36E-02 | ['PLAT', 'SELP', 'IL6'] |
| **STRING DISEASES** | Lung disease | 3.03E-05 | 3.36E-02 | ['PLAT', 'RET', 'IL6', 'TGFA'] |

**Pathway enrichment analysis of the top 20 biomarkers associated with circulating 5-oxoproline.** Enrichment was performed in Enrichr (Gene Ontology Biological Process, Molecular Function, and Cellular Component [2023]; KEGG [2021 Human]; Reactome [2022]; WikiPathways [2023 Human]; MSigDB Hallmark [2020]) and the STRING database (DISEASES). For each database the top-ranked terms are shown (up to 10; all five returned terms for STRING DISEASES). P-values are from Fisher's exact test; Adjusted_P is the Benjamini-Hochberg FDR. Genes lists the input proteins mapped to each term. The WikiPathways term “EGFR Tyrosine Kinase Inhibitor Resistance (WP4806)” is referred to in the main text as EGFR tyrosine kinase signaling.

**Table S6. Targeted post hoc re-analysis of circulating proteins versus 5-oxoproline.**

| **Protein** | **Pearson r (95% CI)** | **Spearman ρ** | **Univariate P** | **BH-adj. P** |
| --- | --- | --- | --- | --- |
| **DCN** | 0.127 (0.059–0.194) | 0.135 | 2.5e-4 | 0.003 |
| **VIM** | 0.124 (0.056–0.191) | 0.119 | 3.7e-4 | 0.004 |
| **TGFBR2** | 0.116 (0.048–0.183) | 0.109 | 8.3e-4 | 0.007 |
| **MMP-9** | 0.112 (0.044–0.179) | 0.105 | 0.001 | 0.010 |
| **TGM2** | 0.104 (0.036–0.171) | 0.095 | 0.003 | 0.017 |
| **KRT19** | 0.094 (0.026–0.161) | 0.079 | 0.007 | 0.032 |
| **OLR1/LOX-1** | 0.088 (0.020–0.156) | 0.085 | 0.011 | 0.044 |
| **ADM** | 0.083 (0.014–0.150) | 0.143 | 0.018 | 0.063 |
| **KIM-1** | 0.034 (-0.035–0.102) | 0.062 | 0.334 | 0.546 |
| **MMP-7** | -0.059 (-0.127–0.009) | -0.039 | 0.090 | 0.217 |
| **EGLN1** | 0.098 (0.030–0.165) | 0.074 | 0.005 | 0.024 |
| **GLO1** | 0.093 (0.025–0.160) | 0.070 | 0.008 | 0.034 |
| **RAGE** | 0.081 (0.012–0.148) | 0.098 | 0.021 | 0.070 |
| **PARP1** | 0.074 (0.006–0.142) | 0.082 | 0.033 | 0.099 |
| **HO-1** | 0.047 (-0.021–0.115) | 0.063 | 0.176 | 0.366 |
| **PRDX1** | 0.043 (-0.025–0.111) | 0.039 | 0.213 | 0.415 |
| **PADI2** | 0.040 (-0.028–0.108) | 0.065 | 0.247 | 0.452 |
| **BACH1** | 0.017 (-0.052–0.085) | 0.043 | 0.633 | 0.787 |
| **PRDX5** | 0.006 (-0.063–0.074) | -0.015 | 0.872 | 0.924 |
| **ARNT** | -0.030 (-0.098–0.038) | 0.020 | 0.385 | 0.587 |
| **SOD2** | -0.036 (-0.104–0.032) | -0.031 | 0.300 | 0.514 |
| **PRDX3** | -0.048 (-0.116–0.020) | -0.061 | 0.165 | 0.349 |
| **Osteopontin/SPP1** | 0.071 (0.002–0.138) | 0.090 | 0.042 | 0.117 |
| **TIMP4** | 0.052 (-0.016–0.120) | 0.067 | 0.137 | 0.304 |
| **Collagen I/COL1A1** | 0.043 (-0.025–0.111) | 0.043 | 0.218 | 0.422 |
| **MMP-12** | 0.020 (-0.049–0.088) | 0.029 | 0.571 | 0.737 |
| **MMP-3** | 0.018 (-0.051–0.086) | 0.004 | 0.610 | 0.768 |
| **SPARC** | 0.015 (-0.053–0.083) | -0.004 | 0.669 | 0.816 |
| **THBS2** | 0.007 (-0.061–0.076) | 0.017 | 0.835 | 0.911 |
| **MMP-2** | -0.017 (-0.086–0.051) | -0.009 | 0.617 | 0.774 |
| **PAI-1/SERPINE1** | -0.022 (-0.090–0.047) | -0.038 | 0.533 | 0.714 |
| **TGF-α** | 0.230 (0.164–0.294) | 0.210 | 2.4e-11 | 8.5e-9 |

Targeted post hoc re-analysis of circulating proteins versus 5-oxoproline. Pearson correlation (r) with Fisher 95% confidence interval (CI), Spearman ρ, univariate P, and Benjamini–Hochberg-adjusted P computed across all 355 panel proteins (n = 823). BH-sig: significant at BH-adjusted P < 0.05. Seven of ten tissue-remodeling/renal-injury proteins and two redox/stress proteins (GLO1, EGLN1) remained significant after full-panel BH correction; nine of ten tissue-remodeling proteins were directionally positive. OLR1/LOX-1 = oxidized-LDL receptor-1 (not lysyl oxidase).
